# Concept-drifts adaptation for machine learning EEG epilepsy seizure prediction

**DOI:** 10.1038/s41598-024-57744-1

**Published:** 2024-04-08

**Authors:** Edson David Pontes, Mauro Pinto, Fábio Lopes, César Teixeira

**Affiliations:** 1 Department of Informatics Engineering, CISUC, University of Coimbra, Coimbra, Portugal; 2 Epilepsy Center, Department Neurosurgery, Medical Center - University of Freiburg, Faculty of Medicine, University of Freiburg, Freiburg, Germany

**Keywords:** Diseases, Health care, Medical research

## Abstract

Seizure prediction remains a challenge, with approximately 30% of patients unresponsive to conventional treatments. Addressing this issue is crucial for improving patients’ quality of life, as timely intervention can mitigate the impact of seizures. In this research field, it is critical to identify the preictal interval, the transition from regular brain activity to a seizure. While previous studies have explored various Electroencephalogram (EEG) based methodologies for prediction, few have been clinically applicable. Recent studies have underlined the dynamic nature of EEG data, characterised by data changes with time, known as concept drifts, highlighting the need for automated methods to detect and adapt to these changes. In this study, we investigate the effectiveness of automatic concept drift adaptation methods in seizure prediction. Three patient-specific seizure prediction approaches with a 10-minute prediction horizon are compared: a seizure prediction algorithm incorporating a window adjustment method by optimising performance with Support Vector Machines (Backwards-Landmark Window), a seizure prediction algorithm incorporating a data-batch (seizures) selection method using a logistic regression (Seizure-batch Regression), and a seizure prediction algorithm with a dynamic integration of classifiers (Dynamic Weighted Ensemble). These methods incorporate a retraining process after each seizure and use a combination of univariate linear features and SVM classifiers. The Firing Power was used as a post-processing technique to generate alarms before seizures. These methodologies were compared with a control approach based on the typical machine learning pipeline, considering a group of 37 patients with Temporal Lobe Epilepsy from the EPILEPSIAE database. The best-performing approach (Backwards-Landmark Window) achieved results of 0.75 ± 0.33 for sensitivity and 1.03 ± 1.00 for false positive rate per hour. This new strategy performed above chance for 89% of patients with the surrogate predictor, whereas the control approach only validated 46%.

## Introduction

Epilepsy is a neurological disease affecting over 50 million people worldwide and is characterised by abnormal brain activity leading to seizures^1–3^. While anti-epileptic drugs (AED) serve as the primary treatment, approximately one-third of patients with Drug-Resistant Epilepsy (DRE) experience uncontrolled seizures, resulting in psychological distress and early death. Although epilepsy surgery offers a solution for DRE, it remains accessible to only a fraction of eligible candidates. In this context, seizure prediction emerges as a crucial tool in clinical management, aiming to enhance the quality of life for individuals vulnerable to sudden seizures.

Traditionally, epilepsy seizure prediction methodologies usually use the EEG signal, requiring models to contend with the inherent complexities of epilepsy, its seizures, and brain dynamics. Researchers typically divide the EEG signals into four periods: preictal, the period that precedes the seizure; ictal, the period corresponding to the seizure; postictal, the period that succeeds the seizure; and ultimately, the interictal, the period found between the postictal and the preictal periods of consecutive seizures^4,5^. The preictal is the most challenging period to detect as there are no effective bio-markers for all patients. The lack of guidelines comes from the fact that a clinician cannot timely predict a seizure by visually inspecting the EEG and that heterogeneity can be found, not only from one patient to another but also between seizures within the same patient^6^.

Machine learning (ML) approaches are widely used in seizure prediction^7–13^. Current seizure prediction algorithms follow a common framework that includes signal pre-processing, feature extraction, feature selection, classification and post-processing. Certain studies used a grid-search over various intervals to choose a pre-ictal interval unique to each patient (e.g. 10, 20, 30, 60, or even 240 min)^14,15^. However, existing methods often overlook the dynamic nature of real-world data, such as the case of EEG, leading to performance degradation over time^16^. Since seizures are rare events, class distributions are often skewed leading to class imbalance. Additionally, the data used is non-stationary where changes in the hidden context or data distribution may occur, leading to concept drifts.

The present work aims to develop EEG-based patient-specific algorithms for epilepsy seizure prediction while addressing concept drifts. The algorithm should be able to retrain itself, recall, or forget information to improve its performance. Our research question centres on devising methods to predict seizures while dynamically adapting to changing contexts during the learning process. To this end, we propose and evaluate three patient-specific seizure prediction approaches against a control method, each incorporating strategies for concept drift adaptation. Specifically, we introduce a seizure prediction algorithm incorporating a window adjustment method by optimising performance with Support Vector Machines^1^ (Backwards-Landmark Window), a seizure prediction algorithm incorporating a data-batch (seizures) selection method using a logistic regression^2^ (Seizure-batch Regression), and a seizure prediction algorithm with a dynamic integration of classifiers^3^ (Dynamic Weighted Ensemble). The models were retrained after each seizure using the last three seizures.

A standard prediction study attempts to simulate real-time by building algorithms that work in a device that receives the EEG and releases alarms, hopefully giving the patient a minimal preparation time^9,17^. Many times, EEG data is acquired when the patient is under pre-surgical monitoring to assess surgery eligibility. In these conditions, medication alteration and sleep deprivation are changes expected, as they are used to provoke seizures. Therefore these studies can only be seen as proofs of concept, where concept drift adaptation is required. Furthermore, in seizure prediction, the underlying context includes daily-life habits, medication, stress situations, the circadian, ultradian, and infradian rhythms, cognitive states, environmental changes, implanting a neurostimulation device, a sudden brain lesion, and others that can affect the brain dynamics and consequently modify optimal characteristics for anticipating seizures^3,5,6,18–20^.

This study explores an attempt to imitate real life by constructing seizure prediction models, using pre-surgical monitoring data. After training, the models were tested chronologically and iteratively in unseen data. Its preictal period spans from 10 to 50 min, with an intervention time of 10 min for each prediction, enough time for the patient to avoid accidents and/or for rescue medication intake^13,21^. Through a rigorous evaluation process, we aim to establish novel methodologies capable of effectively predicting seizures, thus enabling timely interventions to mitigate their impact on individuals with epilepsy.

### Concept-drifts adaptation related works

The adaptation to concept drifts in machine learning involves addressing two main challenges: detecting the occurrence of concept drift and adjusting predictions based on the new data. Various questions arise when developing techniques to handle concept drift, such as how data is processed, how learning tasks are conducted, how concept drift is monitored, and how it is ultimately addressed. Techniques for addressing concept drift include modifying the training set, employing adaptive base learners, and using ensemble methods^16,22^. Modification approaches, such as sequential methods and windowing techniques, focus on selecting or weighing instances seen by the classifier to adapt to changing data distributions^16,22,23^. Single learners dynamically adapt to new training data batches, while ensemble learners maintain diversity and adaptability by managing training sets, base learners, and final decision processes^3,22,24^.

The monitoring process for concept drift involves using supervised or unsupervised indicators based on the availability of predictive feedback^3,22,23^. Supervised indicators are suitable when true labels are instantly accessible, while unsupervised indicators are preferred when prediction feedback is delayed or only partially labelled. Finally, adapting to concept drifts involves either informed methods, which detect drift through triggering mechanisms, or blind systems that implicitly adapt to changes without explicit drift detection^22,25^. These techniques collectively offer a comprehensive framework for addressing concept drift in machine learning systems, with each aspect requiring careful consideration and implementation for effective drift adaptation.

## Materials and methods

**Figure 1:**
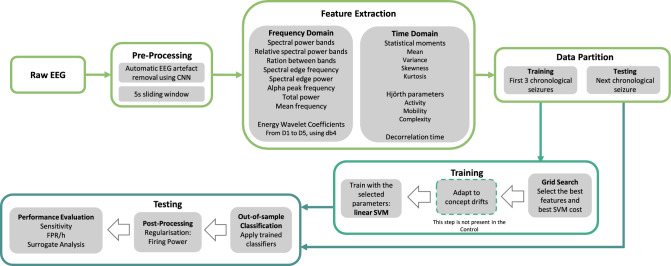
Flowchart of the proposed seizure prediction for each patient, comprising data processing, feature extraction, training, testing and performance evaluation.

Since we created a patient-specific strategy, we applied the following procedure to each patient: data pre-processing, feature extraction, training, and testing (see Fig. 1). After pre-processing, we extracted univariate linear features from time and frequency domains. We developed three seizure prediction pipelines to intrinsically adapt to concept drifts, they were iteratively trained, by accumulating data from previous seizures and tested on the following one. The Backwards-Landmark Window aims to select the samples from and close to the current concept. The Seizure-batch Regression tries to choose the best combination of past seizures to train the classifier. The Dynamic Weighted Ensemble builds a dynamically integrated ensemble to recall older concepts. Finally, we applied the models to the testing set, and to evaluate their performance, accounted by sensitivity and FPR/h. We also performed Statistical validation with surrogate time series analysis^26^. Finally, we tuned this methodology on a machine equipped with an Intel Core i7-8700 3.20GHz processor, 30GB of RAM running on Windows 10 and using Python 3.7 on Spyder 4.0.1. The used Python libraries were: *numpy, pandas, pickle, datetime, time, matplotlib, seaborn, scipy, random, math,* and *sklearn*.

### Data

For the present study, we selected from the European Epilepsy Database, developed on behalf of the FP7 EPILEPSIAE project (www.epilepsy-database.eu)^27^, 37 DRE patients (16 female and 21 male, with a mean age of 40.57 ± 15.76 years). The chosen EEG data were gathered from patients with seizures in the temporal lobe by the University Medical Centre of Freiburg in Germany. It covers 19 EEG electrodes placed according to the International 10–20 System obtained at a sampling rate of 256 Hz. We chose the 37 patients based on the number of independent seizures. We selected patients with at least four lead seizures spaced by at least 4.5h. Consequently, we considered 207 of the 350 seizures appropriate for analysis, resulting in a total recording duration of 5125 h ($\approx$ 7.12 months). We collected all the analysed data while patients were at the clinic under pre-surgical monitoring. The use of this data has been approved for research purposes by the Ethical Committee of the three hospitals involved in the development of the database (Ethik-Kommission der Albert-Ludwigs-Universität, Freiburg; Comité consultatif sur le traitement de l’information en matière de recherche dans le domaine de la santé, Pitié-Salpêtrière University Hospital; and Comité de Ética do Centro Hospitalar e Universitário de Coimbra). All methods were performed following the relevant guidelines and regulations. Informed written patient consent from all subjects and/or their legal guardian(s) was also obtained. More information regarding the selected patients can be found in the Supplementary material.

### Pre-processing and feature extraction

We segmented all patient data into 5-second non-overlapping windows, filtered, and with artefacts minimised by a model based on CNNs. Lopes et al.^28^ developed this model to automatically minimise artefacts from EEG signals in a manner comparable to that of experts.

Next, for each time window in each electrode, we extracted 59 univariate linear features used in the literature^11,13,14,29–34^ which are computationally light. See the Supplemental Material in the Feature Description section to understand each feature’s a priori expected perceived quality. It is also crucial to remember that a variety of nonlinear and bi/multivariate features would also be interesting to explore^19,32,35,36^. However, this would significantly increase the computational effort of our work. We extracted the following features from the frequency domain: relative spectral power bands delta (0.5–4Hz), theta (4–8Hz), alpha (8–13Hz), beta (13–30Hz), four gamma sub-bands - gamma band 1 (30–47Hz), gamma band 2 (53–75Hz), gamma band 3 (75–97Hz), and gamma band 4 (103–128 Hz), the ratio between these bands, spectral edge frequency and power, alpha peak frequency, total power, mean frequency, and the energy of the wavelet coefficients (from D1 to D5, using the db4 mother wavelet). As for the time domain, we extracted the following features: the four statistical moments (mean, variance, skewness, kurtosis), Hjörth parameters (activity, mobility, complexity), and decorrelation time. It is worth mentioning that a consensus among authors regarding these sub-bands cannot be found. Additionally, as a frequency limit of gamma activity is bounded by half of the sampling frequency, and its division into high-gamma and low-gamma is not uncommon^37^, we decided to divide it into four sub-bands. Moreover, given that these recordings are extra-cranial, the gamma band powers may most likely include muscle artefacts.

### Classifier training

We chose the SVM as it is widely used in the literature^8,11,14,29–31,33,35,38^. Also, the SVM is appealing from the perspective of interpretability due to its ability to linearise the feature space and analyse the produced support vectors. However, it is essential to remember that (as with any other classifier) its interpretability may be lost if the number of features becomes excessive.

**Figure 2:**
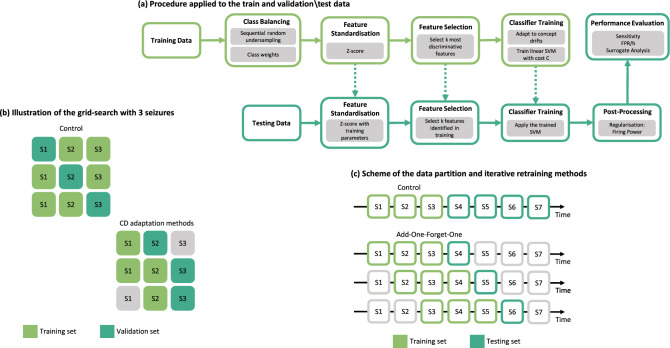
An illustrated scheme of the iterative retraining and validation\testing in the algorithm input seizures.

Figure 2 illustrates how we evaluated the models. We performed this iteratively by retraining the SVM-based classifier after each new seizure, using metrics commonly adopted in seizure prediction. Validation seizures are those used in the grid-search to assess and validate the model from the seizures chosen to train the algorithms. After training, we used the same procedure to test new seizures, referred to as testing seizures (Fig. 2.b). Thus, for each validation seizure, we choose the classifier hyper-parameters, such as the SVM cost, the number of features, and the appropriate preictal duration. Using two measures, sample sensitivity $S_{ss}$ (ratio of samples identified as pre-ictal within all preictal samples) and sample specificity $S_{sp}$, we assessed the seizure prediction models throughout the grid-search (ratio of samples classified as interictal within all interictal samples). Then, we standardise the feature set with z-scoring.

We trained a classifier using the standardised features, and as seizures are very rare, there is a considerable imbalance between interictal and preictal classes. To balance the samples’ classes, during the training phase, we conducted a systematic random undersampling for the Control approach and used class weights inversely proportional to their frequency of occurrence for the methods able to adapt to concept drifts.

We retrained the models using only three seizures. This is, incrementing seizure by seizure and removing the oldest training one. We named this method Add-One-Forget-One. Lopes et al.^39^ also periodically retrained their models but with a slightly different method. They also retrained after each seizure but kept all the previous ones for training. They named this method Chronological. We conducted an additional comparison between the different methods, yielding supplementary data that further support the decision to opt for the Add-One-Forget-One method. Detailed results are available in the complete thesis^40^ and the Supplementary Material.

#### Concept-drifts adaptation

**Figure 3:**
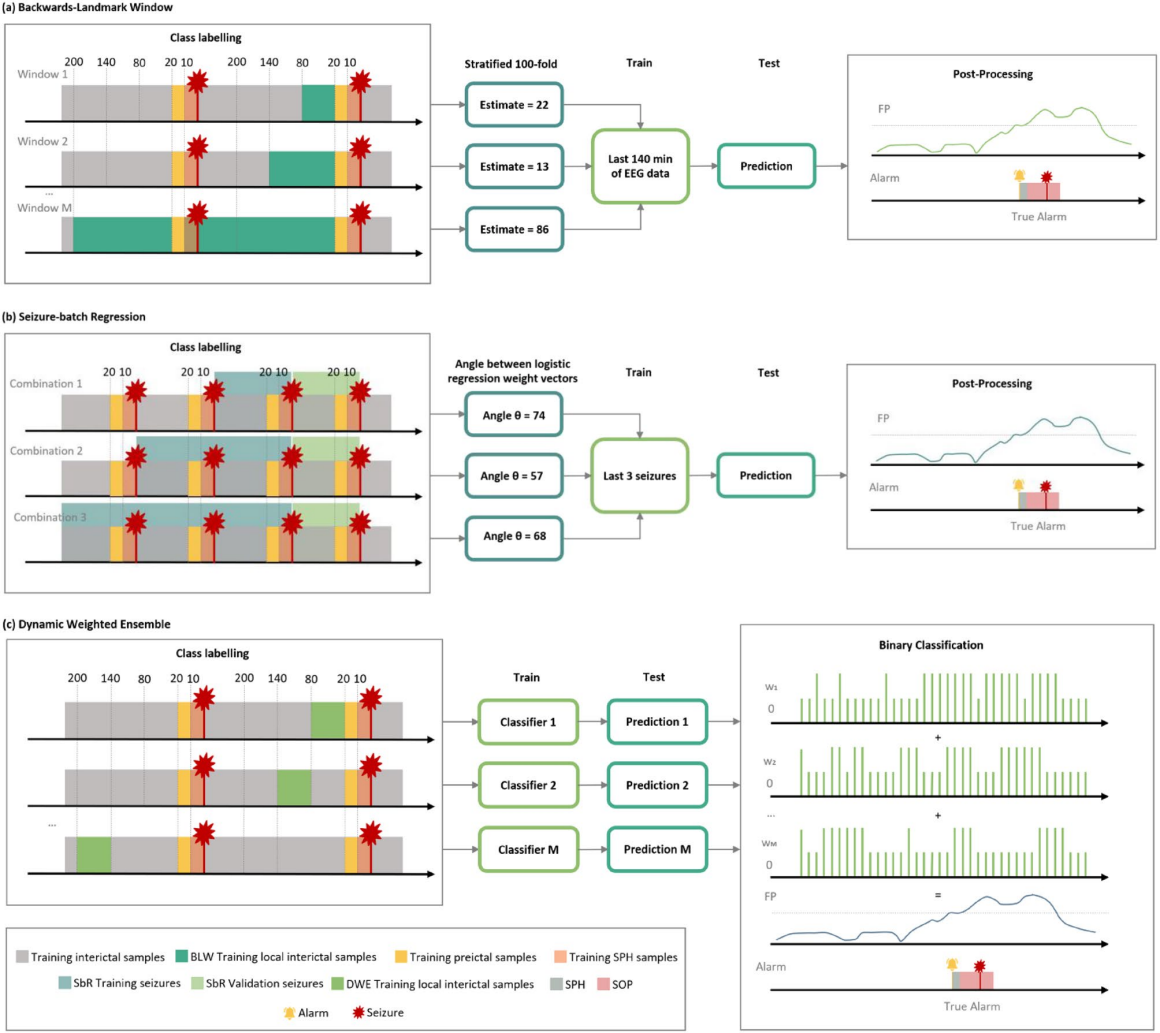
Examples of the algorithm outputs for each of the three proposed concept drift adaptations: (**a**) Window size #2 would be the one selected as it has the smallest window estimate with a value of 10; (**b**) The selected combination of past seizures would be the second one as it possesses the smallest angle with a value of 57^∘^; (**c**) We gave each classifier a weight proportional to its accuracy in the last two hours before the last training seizure. Then, we integrated the classifiers using weighted voting.

Regarding concept drift adaptation, we proposed three different approaches to predict seizures in the present work (see Fig.3). We modified the most common seizure prediction methodology to implement Backwards-Landmark Window, Seizure-batch Regression, and Dynamic Weighted Ensemble. Each approach is further detailed in this section.

*(a) Backwards-Landmark Window.* We developed the Backwards-Landmark Window to create an algorithm that can select the data associated with the concept more similar to the preictal period of the last labelled seizure. With that in mind, a window adjustment algorithm was employed. The algorithm had to solve the trade-off between larger windows providing more data, and thus, more generalising ability, and changes in the concept. We used a window adjustment algorithm adapted from Klinkenberg and Joachims^1^ to solve this problem. At batch *t*, this algorithm tries various window sizes of 1-hour difference, training a SVM for each resulting training set. For each window size, it computes a leave-one-out estimate. The algorithm selects the window size that minimises the leave-one-out estimate of the error rate. Refer to the Supplementary Material for more details on the algorithm.

*(b) Seizure-batch Regression.* Unlike the previous approach that monitors concept drifts by tracking previous samples, Seizure-batch Regression does it by selecting the seizures (data batches) more relevant to learn the current concept. We adapted this approach from Yeon et al.^2^, and it aims to discover the best combination of past seizure data. The algorithm trains a logistic regression for each chronological combination of train seizures and compares its weights with a logistic regression trained only with the last train seizure. That is done by calculating an angle between the two logistic regression weight vectors for each combination. The angle between the vectors is defined by:

1 $$\begin{aligned} \theta ({\vec {w}_{1}},{\vec {w}_{2}})= cos^{-1}\left( \frac{{\vec {w}_{1}} \cdot {\vec {w}_{2}}}{||{\vec {w}_{1}}||\times ||{\vec {w}_{2}}||}\right) \end{aligned}$$

Where $\theta (w_{1},w_{2})$ is the angle between the vectors $w_{1}$ and $w_{2}$. The combination selected is the one with the smallest angle. Because when $\theta (w_{1},w_{2})$ is small, we assumed there is no drift or a gradual concept drift, and when $\theta (w_{1},w_{2})$ is large there is considerable concept drift. Refer to the Supplementary Material for more details on the algorithm.

*(c) Dynamic Weighted Ensemble.* We developed this approach because ensembles are among the most popular and effective techniques to handle concept drifts^3^. Here, we kept a set of models built over different 1-hour periods and combined the models’ predictions according to their expertise level regarding the current concept. We assumed that the two hours before seizure onset were the current concept. In this work, we employed a dynamic integration of SVM classifiers. Each base classifier is given a weight proportional to its accuracy in the last two hours before the last training seizure. Then we integrated the classifiers using weighted voting. Refer to the Supplementary Material for more details on the algorithm.

### Testing, statistical validation, and performance comparison

For each patient, we ran each algorithm and tested the resulting models using unseen data. Then, using all the parameters (mean and standard deviation for z-scoring) and models learned during training, we apply a similar procedure to the testing seizures. The classifier output was then smoothed and strengthened against noise using the Firing Power regularisation technique^41^. The latter functions as a moving-average filter and sets off an alarm when a threshold is surpassed. We decided to exclude the Firing Power threshold from the grid-search since it would be another parameter to tune, therefore extending computation time. As a result, we established 0.5 (half of the scale that is between 0 and 1) as a fair value without any adjustment.

Additionally, in the testing phase, we introduced after each raised alarm, a time frame with a duration equal to the chosen pre-ictal time, during which no more alarms are allowed, called refractory period (see Fig. 2.a). To ensure that we estimate performance metrics correctly, we removed these periods from the False Positive Rate per Hour (FPR/h) calculation^18^. Then, we conducted a Surrogate analysis to determine whether a given approach is performing above chance^12,13,26^. We moved the initial seizure onset times to a random position in the interictal interval. We did it seizure by seizure to ensure that the simulated seizure times respected the seizure distribution across time. We then calculated the sensitivity using the surrogate times (new labels). The average sensitivity that resulted from running this process 30 times was compared to the obtained sensitivity using the proposed methods. When our algorithm’s sensitivity is higher with statistical significance regarding the surrogate, we considered it to have achieved a performance above chance level. We tested the following null hypothesis using a one-sample t-test with a statistical significance level of 0.05: “The sensitivity of the suggested approach is not superior to the sensitivity of the surrogate predictor.”

## Results

This section presents the results obtained in the present study and their interpretative analysis. The first subsection is focused on the proposed approaches to intrinsically adapt to concept drifts during the learning process. In contrast, the second subsection concentrates on understanding how the models and the preictal period evolve after each seizure.

### Concept drift adaptation

**Figure 4:**
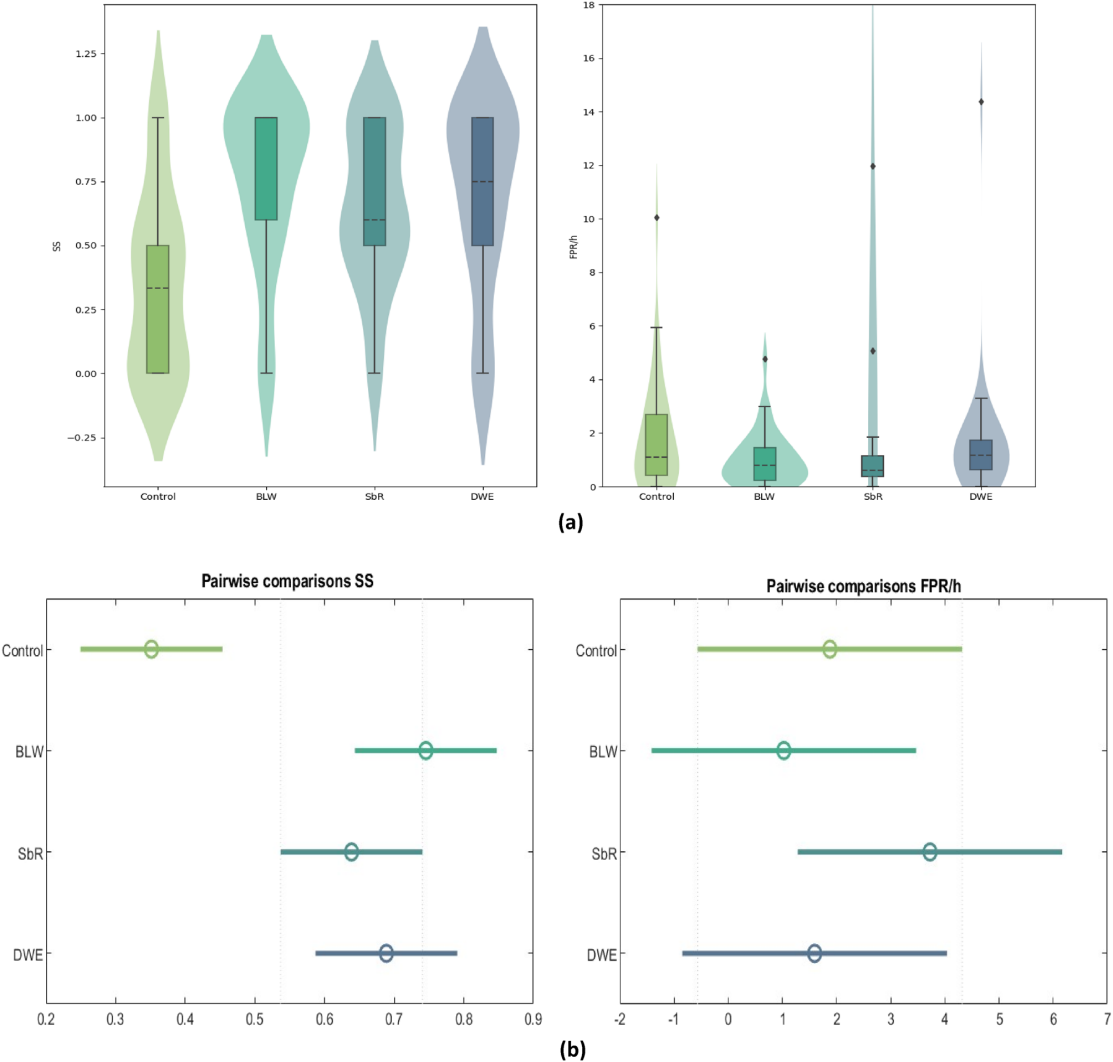
Performance comparison between approaches. BLW stands for Backwards-Landmark Window, SbR for Seizure-batch Regression, and DWE for Dynamic Weighted Ensemble.

Table 1 shows the performance of the adopted methodologies. Figure 4a displays violin plots with boxplot overlays illustrating overall seizure sensitivity (SS) and FPR/h values for all approaches. Figure 4b summarises the results obtained for pairwise statistical comparison. The average sensitivity values of the proposed approaches in this study are generally very similar. However, they exhibit substantial standard deviations, indicating that although some patients’ models could be strong predictors, others may perform poorly. Observing the violin plots for SS values (Fig. 4a ) we can notice a large dispersion of sensitivity values but are restricted for approximately 75% of the patients on all approaches to values above 0.4, except for the Control. Analysing the results obtained in the pairwise comparisons for seizure sensitivity (Fig. 4b), we can conclude that the three proposed methods show SS values significantly higher than the Control.

**Table 1:** Average seizure prediction performance across all patients for each approach.

Approach	All patients			Validated patients		
	SS	FPR/h	SS surrogate	%	SS	FPR/h
Control	0.35 ± 0.35	1.88 ± 2.05	0.25 ± 0.50	45.95	0.62 ± 0.28	1.75 ± 1.66
Backwards-landmark window	0.75 ± 0.33	1.03 ± 1.00	0.22 ± 0.47	89.19	0.81 ± 0.25	0.91 ± 0.81
Seizure-batch regression	0.64 ± 0.31	3.73 ± 15.82	0.23 ± 0.48	86.49	0.68 ± 0.27	0.67 ± 0.51
Dynamic weighted ensemble	0.69 ± 0.36	1.60 ± 2.26	0.25 ± 0.50	83.78	0.79 ± 0.28	1.18 ± 0.76

However, it is also noticed that outliers (e.g., 10.04, 4.77, 5.07, 11.96, and 14.37) had a distinct and significant negative impact on the average values of FPR/h (refer to the Supplementary Material). The Backwards-Landmark Window and the Dynamic Weighted Ensemble comprise lower FPR/h values in comparison with the Control. The Seizure-batch Regression has an outlier with an FPR/h of 97.86, which, if removed, gives the Seizure-batch Regression a smaller average FPR/h than the Control. Nevertheless, the seizure prediction models from this study raised many false alarms, making them unsuitable for a warning system. The average sensitivity of the three proposed approaches transcended the one from surrogate time series analysis. All approaches statistically validated more than 80% of the analysed patients. With the Backwards-Landmark Window, 33 out of 37 patients (89.19%) performed above the chance level. Moreover, as expected, the metrics derived for the group of validated patients were significantly better than those for all patients. But even for these patients, the FPR/h values are generally higher than the desirable. The Backwards-Landmark Window may be the best approach for the 37 studied patients, as it had the highest sensitivity (0.75 ± 0.33), lowest FPR/h (1.03 ± 1.00), and the most significant percentage of statistically validated patients.

**Figure 5:**
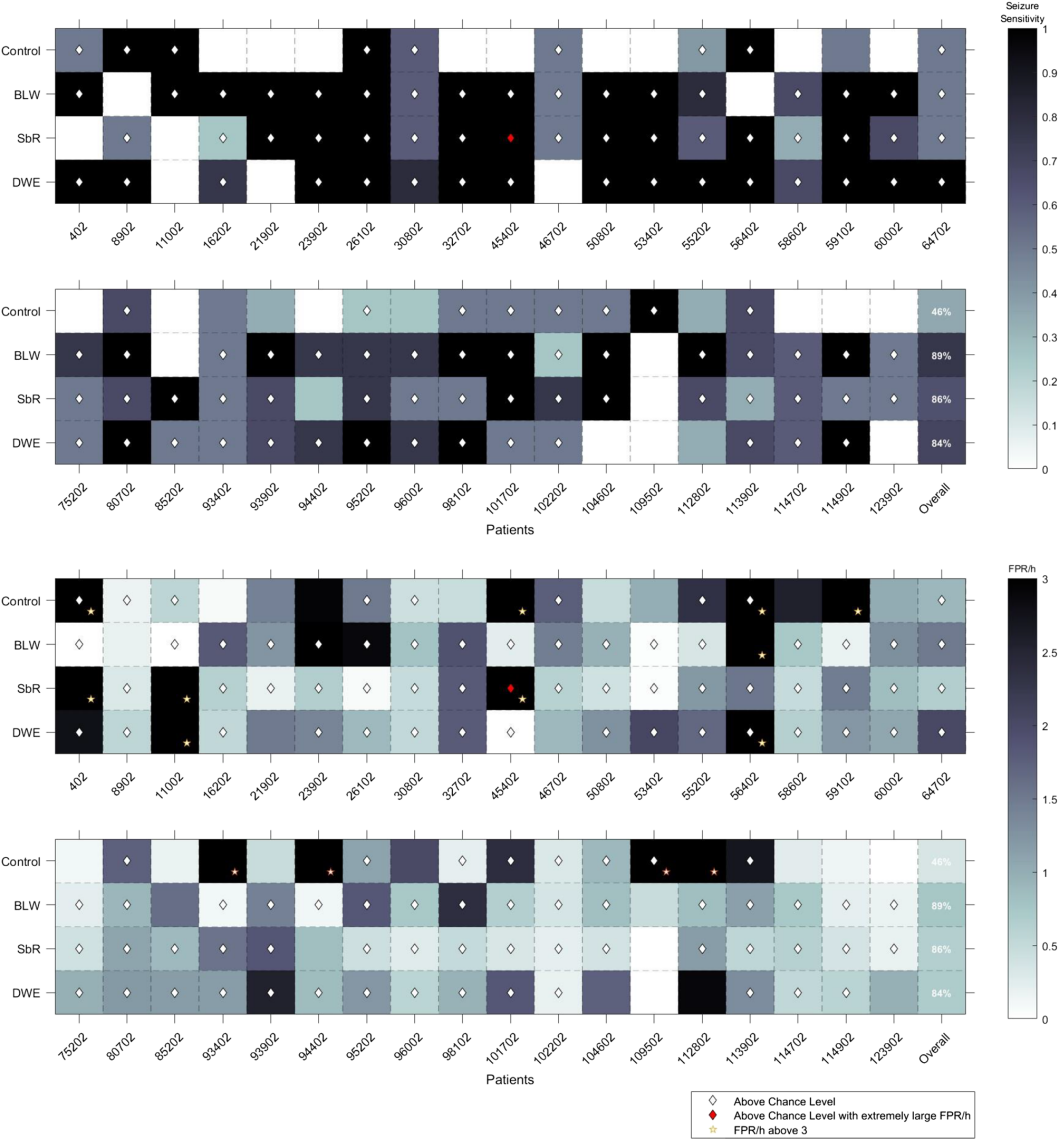
Sensitivity and FPR/h of seizure prediction and statistical validation results for the proposed approaches for each patient. The blue colour scale refers to the sensitivity achieved, the white diamond shape is present when statistically validated, and the red diamond shape when the performance is above chance level, but not considered statistically validated because of the large FPR/h. On the overall column, for each approach, one can see the percentage of patients whose models performed above the chance level along with the average sensitivity given by the colour of the cell. BLW stands for Backwards-Landmark Window, SbR for Seizure-batch Regression, and DWE for Dynamic Weighted Ensemble.

In Fig. 5, we illustrate a comparison of the achieved performances for each patient and for each of the proposed methodologies. Ten (27%) patients, performed above chance level for all approaches. Additionally, 54% of the patients (16202, 21902, 23902, 32702, 45402, 50802, 53402, 58602, 59102, 60002, 75202, 85202, 93402, 93902, 94402, 96002, 112802, 114702, 114902, 123902) achieved performance above the chance level for at least one of the approaches that adapted concept drifts but not with the control approach.

Table 2 shows the test results for the set of patients and patient stratification. We based our stratification on seizure classification (focal onset aware seizures (FOA) or focal onset impaired awareness seizures (FOIA) only), seizure activity (only rhythmic) and vigilance state at seizure onset (awake only). The table shows that the percentage of validated patients only improved for the Control in the FOA or FOIA seizure-stratified groups. For the second group, composed of patients with rhythmic activity patterns in their seizure, the ratio of validated patients was higher for the Control and the Backwards-Landmark Window. For the group of patients that only had seizures while awake, the percentage of validated patients was higher only for the Dynamic Weighted Ensemble.

**Table 2:** Test results for the overall set of patients and stratified sets of patients.

Approach	Stratification	Number of patients	SS	FPR/h	Validated patients
Control	Only FOA or FOIA seizures	25	0.35 ± 0.36	1.94 ± 2.06	44.00%
	Rhythmic activity only	36	0.36 ± 0.35	1.93 ± 2.06	47.22%
	Awake-only onset seizures	16	0.45 ± 0.38	0.38 ± 2.61	50.00%
	Overall	37	0.35 ± 0.35	1.88 ± 2.05	45.95%
Backwards-landmark window	Only FOA or FOIA seizures	25	0.73± 0.33	0.94 ± 0.84	88.00%
	Rhythmic activity only	36	0.74 ± 0.30	0.92 ± 0.98	91.67%
	Awake-only onset seizures	16	0.76 ± 0.38	1.33 ± 1.30	81.25%
	Overall	37	0.75 ± 0.33	1.03 ± 1.00	89.19%
Seizure-batch regression	Only FOA or FOIA seizures	25	0.65 ± 0.31	4.95 ± 19.10	84.00%
	Rhythmic activity only	36	0.63± 0.26	0.69 ± 0.50	86.11%
	Awake-only onset seizures	16	0.64 ± 0.34	7.14± 23.45	81.25%
	Overall	37	0.64 ± 0.31	3.73 ± 15.82	86.49%
Dynamic weighted ensemble	Only FOA or FOIA seizures	25	0.66 ± 0.35	1.59 ± 2.69	80.00%
	Rhythmic activity only	36	0.70 ± 0.33	1.31 ± 0.78	83.33%
	Awake-only onset seizures	16	0.84 ± 0.29	1.26 ± 0.97	87.50%
	Overall	37	0.69 ± 0.36	1.6 ± 2.26	83.78%

### Concept drift adaptation analysis

To understand how the models and the preictal period change after each seizure, we evaluated the SVM cost, features, scalp EEG channels and SOP duration for the best approach (Backwards-Landmark Window). For this reason, we calculated the relative frequencies of the selected parameters over all patients. In general, we could not draw any conclusions. Therefore, we chose the three patients (30802, 55202, 114702) with eight seizures and analysed them. The relative frequencies of the set parameters over all patients and for 55202 and 114702 can be found in the Supplementary material.

**Figure 6:**
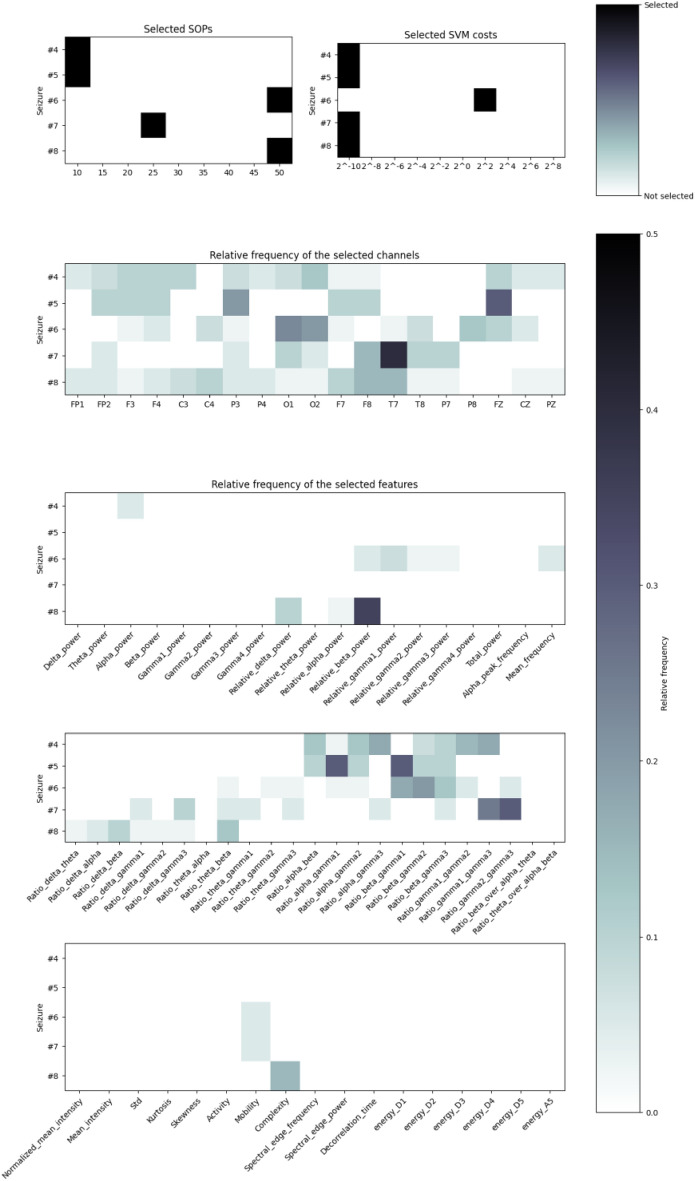
Relative frequency of the selected SOP duration, SVM costs, channels, and features for patient 30802.

For example, in patiet 30802 we noticed a clear preference for the SVM cost of $2^{-10}$, but the preictal duration after the 5th seizure shifts from 10 to 50 minutes. Nevertheless, the number of selected features varied, but there was a preference for 40. We also verified that for this patient, the algorithm selected mostly ratio-related features (see Fig. 6). Initially, the alpha, beta, and gamma bands showed more importance, but after the 6th seizure, the delta, theta, and gamma were more important. Also, some Hjörth parameters were selected from the 6th seizure onward. Regarding the electrode selection, all of them were chosen, ones more frequently than others.

## Discussion

We present the discussion in several subsections: (i) the concept drift adaptation performance and a comparison with the control method, (ii) a comparison with other studies, and (iii) the study limitations.

### Concept drift adaptation

The results suggest that accounting for changes in context within the interictal period while retraining the model after each seizure increases the classifier’s performance. We assume this because the two best-performing approaches (Backwards-Landmark Window and Dynamic Weighted Ensemble), are the ones that adapt to changes of context within the interictal period of each seizure. In contrast, the Control and Seizure-batch Regression do not account for context changes within seizures, only between them. Furthermore, running one execution of the proposed approaches takes approximately 1h6min, 1h16min, and 2h20min for the Backwards-Landmark Window, Seizure-batch Regression and Dynamic Weighted Ensemble, respectively. Since the running time of each of the three proposed methods is shorter than the examined inter-seizure independence period (4h30min), we can consider all for real-life applications.

Additionally, we chose a 10-minute intervention time to grant the patient enough time to take necessary measures for an incoming seizure^42^. This time frame is also suitable for administering diazepam rectal gel, which takes 5-10 minutes to start working.

We also considered essential to discuss the obtained preictal periods and how the classifiers evolved. The relative frequencies of the set parameters over all patients suggest that shorter SOPs may be more frequent (10 and 15 minutes), but it is also noticeable a relatively high frequency for SOPs of 50 minutes. There could be a propensity for the algorithms to choose long SOPs in an attempt to combat class imbalance, as the preictal period has significantly fewer samples than the interictal. Long SOPs are consistent with what has been observed in the literature; hence, there are increasingly more studies with longer SOPs. But does it make sense, given an application or the patients’ will? Patients prefer shorter SOPs since they have less impact on their day-to-day activities when a seizure is imminent^43^.

We could not find a particular subset of more discriminatory features across all patients, but the delta, alpha, and gamma frequency bands appear to be more important. It is important to note that the scalp EEG may not completely capture gamma rhythms, the relatively high frequency of gamma band features may cause some scepticism due to the possible presence of muscle artefacts. These findings lead us to believe that the patients may have muscular jerks due to pre-seizure dynamics recorded by gamma-related features.

### Comparative analysis with other studies

After analysing the results, we compared them with other studies that used scalp EEG data from the EPILEPSIAE database to develop seizure prediction models^7,11–13,39,44^. We only make the comparison with studies from the same database as ours, as it is a more reliable comparison because in seizure prediction the performances vary considerably depending on the database and the type of data. There are some studies^8,31,35^ who also used the EPILEPSIAE database, which cannot be directly compared to our methodology, as model selection was based on the test performance, which is a priori unknown, maybe resulting in an overestimation of the performance.

We chose seven studies that used the EPILEPSIAE database and applied statistical validation to compare the results of our seizure prediction pipeline. The study from Alvarado-Rojas et al.^7^ used 53 patients and is comparable to ours, as the models were not selected based on tested results. They used a threshold classifier, which is intuitive, despite the use of features that may be challenging to comprehend from a clinical perspective. Their results outperformed ours in FPR/h ($\approx$ 0.33), but ours was better in terms of seizure sensitivity ($\approx$ 0.66) and performances above chance level (7/53 $\approx$ 13.21%). Direito et al.^11^ reported a seizure sensitivity of 0.39 and an average FPR/h of 0.21. The percentage of patients performing above chance level was 11.11%. They achieve those results by applying a simple preprocessing method using digital filtering and developed a seizure prediction model based on multiclass SVM using handcrafted features. They also used firing power regularisation with a threshold of 0.5 to smooth the output of the classifiers. Nevertheless, it is worth noting that, both previously mentioned studies, validated their models using the analytical random predictor instead of the surrogate time series analysis. On the other hand, the studies from Pinto et al.^12,13,44^ and Lopes et al.^39^ validated their models using the surrogate time series analysis. Pinto et al.^12,13,44^ published three papers with seizure prediction models with a different number of patients analysed in each. In the first^12^ and second^13^ studies, the algorithms were trained following a chronological approach, and the preprocessing was simple, as it only used digital filters. In the third^44^ study, the preprocessing was done the same way as in this paper, using a model based on CNNs^28^. In this paper, we used data from some patients who were also included in Pinto et al. studies. In the first study^12^, they used data from 19 patients and achieved an average seizure sensitivity of 0.38 ± 0.19 and an average FPR/h of 1.03 ± 0.84, using an SOP of 40 minutes. Performance above chance level was obtained for 32% of the patients. In the second study^13^, they used data from 93 patients and obtained an average seizure sensitivity of 0.16 ± 0.11 and an average FPR/h of 0.21 ± 0.08. 32% of the patients obtained performance above chance level. Both studies had an SPH of 10 minutes. In the third study^44^, they used data from 40 patients and obtained an average seizure sensitivity of 0.17 ± 0.28 and an average FPR/h of 0.87 ± 1.11. Performance above chance level was obtained for 17.50% of the patients. The three studies used firing power regularisation with a threshold of 0.7. Lopes et al.^39^, used data from 41 patients and used the same artefact removal method^28^ as in this study and Pinto et al.^44^. They developed deep neural networks using EEG time series and shallow neural networks using widely-used handcrafted EEG features. Their models were retrained over time and obtained an average seizure sensitivity of 0.37 ± 0.36 and an average FPR/h of 0.86 ± 0.77. Performance above chance level was obtained for 54% of the patients.

Similar to the proposed methodology, the studies under comparison tested a range of SOP values and selected for each patient the best duration, except Lopes et al.^39^, they used an SOP of 30 minutes because it falls within the optimal range of SOPs observed in previous findings, but also since it is sufficiently short to avoid causing anxiety in patients^39,45^. Pinto et al.^12,13,44^ and we selected them based on a specified training metric. Additionally, Direito et al.^11^ and Alvarado-Rojas et al.^7^ took into account shorter seizure prediction horizons of 10 and 60 seconds, respectively, which are inappropriate for a warning system as they do not give the patient enough time to take preventative measures. We consider it important to note that comparing studies becomes difficult when there is high variability in the chosen patient population and the large variety of available parameters and alternatives incorporated throughout the process.

### Study limitations

The present study has some limitations that should be addressed. The first limitation concerns the implementation of an early-stopping mechanism in the Backwards-Landmark Window to reduce computation time; this way, during the search for the more adequate window size, if the window estimate did not improve during the last 12 hours of recordings, the algorithm would stop. Stopping the search before going through the entire dataset is a limitation because there is no way of knowing that a window with a better estimate would not appear. Also, we reasonably set many parameter values, such as the 1-hour window step for the Backwards-Landmark Window and the 1-hour window size for Dynamic Weighted Ensemble, based on the computation time.

Additionally, on average, we tested 2.59 seizures per patient, and 7 of the 37 patients only had one seizure for testing. That is a small number but that is related to the fact that specific models are usually made for each patient. For patients with only one testing seizure, the seizure sensitivity is limited to 0 (seizure not predicted) or 1 (seizure predicted). These differences in potential seizure sensitivity values are responsible for the large standard deviations, which may affect statistical comparisons between patients.

Moreover, the EEG data used is from presurgical monitoring circumstances. Patients in clinics experience medication withdrawal and sleep deprivation, which might lead to more seizures that may not be characteristic of everyday life. Therefore, this study has to be reproduced using ultra long-term recordings made throughout the patients’ daily lives, such as the data from the Neurovista, to access seizure prediction performance fully.

## Conclusion

This work contributed to epilepsy seizure prediction by providing a complete pipeline for patient-specific prediction models while addressing the influence of concept drifts. To this end, we proposed and compared to the Control three seizure prediction approaches (Backwards-Landmark Window, Seizure-batch Regression, and Dynamic Weighted Ensemble) able to adapt to concept drift.

Overall, the proposed approaches that integrated concept drift adaptation techniques outperformed the Control, evidencing the relevance of adapting to concept drifts to predict seizures. In particular, the best-proposed methodology (Backwards-Landmark Window) achieved 0.75 ± 0.33 for sensitivity, 1.03 ± 1.00 for FPR/h, and 89.19% of the patients were statistically validated. This approach aimed to detect the data associated with the last concept before the last labelled seizure. Therefore, taking into account changes in the hidden context over time may be the path to follow in the seizure prediction.

The FPR/h values in this study are inappropriate for real-world warning systems. Still, depending on the effects on the patient’s health, their application in intervention systems (such as electrical stimulation) might be. However, further research must be done to determine the highest FPR/h deemed appropriate for each intervention technique. The proposed pipeline showed a significant performance improvement, but it can only be considered a proof of concept because the data used was in presurgical monitoring, meaning there was a shorter inter-seizure time. Under normal conditions, patients with pharmacorefractory focal epilepsy have a mean seizure frequency of about three seizures per month^42^. Thus, in those situations adopting the Add-One-Forget-One method to retrain the model would mean that approximately one month of recordings would be used. That would significantly increase the inter-seizure time and the model training time. Therefore, to determine its relevance in a real-world scenario, ultra long-term data^46–48^ gathered from conditions encountered in daily life must be used. And only then can it be inferred whether the training time is still inferior than the inter-seizure time, making it applicable in a medical device. Also, only then can it be evaluated whether parameters such as the window step or size should remain 1 hour or be increased to hours or even days.

Concerning future work, the use of Ultra long-term data with naturally occurring seizures, like the one published by Cook et al.^46^, would be extremely important for developing and validating novel prediction algorithms, advancing wider clinical acceptability, and evaluating current methodologies.

### Supplementary Information

Supplementary Information.

## Data Availability

All code is available for public use in: https://github.com/EDavidP/Concept-Drifts-Adaptation-For-Machine-Learning-EEG-Epilepsy-Seizure-Prediction.
