# Supplementary material for: Concept-drifts adaptation for machine learning EEG epilepsy seizure prediction
Source: Sci Rep. 2024 Apr 8;14:8204. doi: 10.1038/s41598-024-57744-1 (PMC11001609; doi:10.1038/s41598-024-57744-1)
Supplement: Supplementary file 1 — Supplementary Information. [file 41598_2024_57744_MOESM1_ESM.pdf]

Concept-Drifts Adaptation For Machine Learning EEG Epilepsy Seizure Prediction

Edson David Pontes[1,*], **Mauro Pinto**[1], **Fábio Lopes**[1,2], **and César Teixeira**[1]

[1]Univ Coimbra, CISUC, Department of Informatics Engineering, Coimbra, Portugal
[2]Epilepsy Center, Department Neurosurgery, Medical Center - University of Freiburg, Faculty of Medicine, University of Freiburg, Freiburg, Germany
[*]edpontes@dei.uc.pt

Supplementary Material

Patient data

Table 1. Information for the 37 studied patients.

Patient ID	Age	Sex	Number of seizures	Seizure classification	Seizure activity pattern	Vigilance at seizure onset	Recording duration (h)
402	55	f	5	FOIA, FBTC, FOIA, FBTC, FOIA	t, t, t, t, t	A, A, A, A, A	133.47
8902	67	f	5	UC, FOIA, FOIA, FOIA, FOIA	a, b, a, m, a	A, A, A, A, A	156.41
11002	41	m	4	UC, FOIA, FOIA, FOIA	?, s, a, t	A, R, A, A	108.86
16202	46	f	7	UC, FBTC, UC, FOIA, FOIA, FOIA, FOIA	r, ?, r, r, r, ?, r	A, A, A, A, A, A, A	235.77
21902	47	m	4	UC, FOIA, FOIA, FOIA	t, t, t, b	A, A, A, R	76.84
23902	36	m	5	FOA, FOA, FOA, FOA, FOA	t, t, t, d, t	A, A, A, A, A	104.69
26102	65	m	4	FOIA, FOIA, FOIA, FOIA	m, t, t, t	A, A, A, A	83.23
30802	28	m	8	FOA, FOA, FOA, FOA, FOA, FOA, FOA, FOA	t ,t ,t, t, t, t, t, t	R, A, 2, A, A, R, 2, 2	149.28
32702	62	f	5	FOIA, FOIA, FOIA, FOIA, FOIA	t ,t ,t, r, a	A, A, A, A, A	141.87
45402	41	f	4	FOIA, FOIA, FOA, FOIA	t, t, t, t	A, A, A, A	94.29
46702	15	f	5	FOA, FOIA, FOIA, FBTC, FOIA	a, a, t, b, t	A, 2, A, 2, A	60.06
50802	43	m	5	FOIA, UC, UC, FOIA, FBTC	t, t, t, t, t	A, 2, 2, 2, A	201.53
53402	39	m	4	FOA, FOA, FOA, FOIA	?, ?, ?, T	A, 2, A, A	84.04
55202	17	f	8	FOIA, FOIA, FOA, UC, UC, FOA, UC, FOIA	t, d, t, t, t, t, r, r	A, A, A, A, A, A, A, A	112.42
56402	47	m	4	UC, UC, UC, FBTC	t, ?, ?, A	A, A, A, A	204.47
58602	32	m	6	FOIA, FOIA, FOIA, FOIA, FOIA, FOIA	r, t, t, r, r, t	A, R, A, A, A, 2	120.28
59102	47	m	5	FOA, FOIA, FOIA, FOIA, FOA	?, t, t, t, t	A, A, A, A, A	148.05
60002	55	m	6	FOIA, FOIA, FOIA, UC, FOIA, FOIA	d, c, t, t, d, d	1, A, A, R, R, 1	360.51
64702	51	m	5	FOA, FBTC, FBTC, FBTC, FBTC	?, m, t, t, t	A, A, A, A, 2	107.5
75202	13	m	7	FOA, FOA, UC, FOA, FOA, FOA, FOA	t, t, t, t, t, ?, t	2, 2, A, A, A, A, A	153.57
80702	22	f	6	FOIA, FOIA, UC, FOIA, FBTC, FOIA	b, b, ?, c, c, c	A, A, A, A, A, A	78.95
85202	54	f	5	FOIA, FOIA, UC, UC, UC	m, c, m, m, m	2, A, A, A, A	73.91
93402	67	m	5	FBTC, FOIA, FOIA, UC, UC	t, t, t, t, t	2, 2, 2, 2, 2	152.07
93902	50	m	6	FOA, FOIA, FBTC, FOIA, FOIA, UC	t, t, d, d, d, d	A, A, 2, A, 2, A	391.12
94402	37	f	7	FOA, UC, FOIA, UC, FOA, UC, FOA	?, d, b, t, ?, b, ?	A, A, A, 2, A ,2, A	150.6
95202	50	f	7	FBTC, FOIA, FOIA, FOIA, UC, FOIA, UC	b, b, b, m, b, b, t	2, 2, 2, 2, 2, 2, 2	147.13
96002	58	m	7	FOIA, FOIA, FOIA, FOIA, UC, FOIA, FOIA	t, t, t, d ,a ,t ,a	A, A, A, A, A, A, A	130.6
98102	36	m	5	FOA, UC, UC, UC, FBTC	?, ?, ?, ?, ?	A, A, A, A, A	154.29
101702	52	m	5	FOIA, FOIA, FOIA, FOIA, FOIA	t, t, t, r, r	A, A, A, 2, A	52.24
102202	17	m	7	FOA, UC, FOIA, UC, FOA, FOIA, UC	b, ?, t, ?, t, t, t	2, A, 2, A, A, 2, A	108.86
104602	17	f	5	FOIA, FBTC, FBTC, FBTC, UC	t, a, t, t, d	A, 2, 2, 2, 2	103.12
109502	50	m	4	FOIA, FOIA, UC, UC	t, t, t, t	A, A, A, A	115.55
112802	52	m	6	UC, FOIA, UC, FOIA, FOIA, UC	t, t, t, t, t, t	A, A, A, A, A, A	183.08
113902	29	f	6	UC, FOIA, FOIA, FOIA, UC, FOIA	t, d, t, t, t, t	A, A, 2, A, 2, A	84.71
114702	22	f	8	FOIA, FOIA, UC, FOIA, FOIA, FOIA, FOIA, FOIA	t, t, t, t, d, t, d, t	A, A, A, A, A, A, A, A	102.43
114902	16	f	7	FOA, FOIA, FOIA, FBTC, UC, FOIA, FOIA	s, b, s, t, r, a, t	A, A, A, 2, A, A, A	77.21
123902	25	f	5	FBTC, FBTC, FOIA, FOIA, FOA	t, t, t, t, t	2, 2, R, A, A	182.26

Gender: female (f), male (m); Seizure classification: unclassified (UC), Focal Onset Aware (FOA), Focal Onset Impaired (FOIA), Focal to Bilateral Tonic-Clonic (FBTC); Seizure activity pattern: unclear (?), rhythmic sharp waves (s), rhythmic alpha waves (a), rhythmic delta waves (d), rhythmic theta waves (t), rhythmic beta waves (b), repetitive spiking (r), cessation of interictal activity (c), amplitude depression (m); Vigilance state: awake (A), REM sleep stage (R), Non-REM sleep stage I (1), Non-REM sleep stage II (2).

Feature Description

Here we provide a more detailed description of the features used in this study. We only used linear univariate features. Linear features are mathematical measures that extract linear dynamics from signals using phase/frequency and amplitude information. When these features are extracted, the EEG signal is considered quasi-stationary within each time window.

Statistical Moments

The Electroencephalogram (EEG) time series amplitude distribution can be determined using statistical moments. The first four statistical moments are the mean, variance, skewness, kurtosis[1–5]. As univariate linear measures are light to compute. They can also be used on characteristics other than the electrical amplitude, such as spectral information[3,6]. For symmetric amplitude distributions, the skewness is zero; for asymmetric distributions, it is non-zero. The kurtosis assesses how peaked or flat an amplitude distribution is[7]. According to Rasekhi et al.[8], these statistical measures have demonstrated considerable changes between the interictal and preictal states. Compared to interictal data, the preictal period showed a decrease in variance and an increase in kurtosis[5].

Hjörth parameters

Hjörth parameters concern activity, mobility, and complexity, which are measures of mean power, root-mean-squared frequency, and root-mean-square frequency spread, respectively. These monitor an increase in brain activity that increases energy[2–5,9]. Authors[8,10] have reported a significant increase of the mobility and complexity of the EEG during the preictal stage.

Decorrelation time

The decorrelation time is defined as the first zero crossing of the autocorrelation function and gives information about the data variability's usual time scale. It estimates the periodicity of data and the strength of linear relationships; the lower its values, the less correlated the signal. This function may also be used to assess signal stochasticity since a temporal value of zero decorrelation indicates that a particular signal is entirely stochastic (white noise). Authors[3,5,8,10] have reported a reduction in decorrelation time before seizures.

Relative spectral power

The signal power associated with particular frequency ranges (delta, theta, alpha, beta, and gamma) is quantified by spectral power. Authors then utilise these ranges to extract features that capture low to high-frequency transitions. These are the most commonly used characteristics[2–5,8,9,11] and may be determined using the Power Spectral Density (PSD). The PSD can be calculated by performing the Fast Fourier Transform (FFT) on the EEG time series and then averaging the squared coefficients of the frequency range of interest. It is critical to note that the PSD calculation presupposes that the signal in each window is short enough to be deemed quasi-stationarity and long enough to capture the brain's low-frequency activity. The power of a specific frequency band divided by the overall power of the EEG signal characterises the relative spectral power. Because there is more power in low frequencies than in high frequencies, normalised spectral power gives a more robust measurement. Some authors documented a power shift from lower to higher frequencies before seizure onset[3,5,8,12]. For example, Mormann et al.[10] demonstrated a drop in Delta band power, accompanied by a fall in relative power in the other sub-bands. Bandarabadi et al.[12] showed that relative combinations of sub-band spectral energies across channel pairs might be used to follow progressive changes before seizures.

Spectral edge frequency

Spectral edge frequency (SEF) is usually referred to as the minimum frequency below which a specified proportion of the total power of the signal is contained, and it is frequently used in seizure prediction[5,8,10]. Most of the spectral strength in the EEG signal is contained in the 0-40Hz band[7]. SEF 50 is the frequency at which 50% of the signal's overall power is situated. As a result, SEF may be able to detect a power shift from low to high frequencies during the preictal phase[3,5].

Wavelet transform

The wavelet transform is a time-frequency domain transform that can be used in place of the FFT. It can show the spectral and temporal features of a signal. The wavelet transform divides the signal into several resolution levels based on the frequency ranges[2,3,5,8]. The first decomposition layers correspond to higher frequencies, whereas the latter levels correspond to lower frequencies. After the signal decomposition, it is possible to compute discriminant features from distinct frequency bands by applying the wavelet coefficients. A characteristic that may be acquired with the wavelet transform is energy measurement in different frequency ranges[3,5,8].

Control method

We used a control methodology to verify the performance of our approach, inspired by the study from Direito et al.[3] used a patient-specific layered structure, which includes filtering, feature extraction and classification. Input signals were filtered by a fourth-order Butterworth Infinite Impulse Response (IIR) from 48Hz to 52Hz to remove the noise related to the power line. The signals were firstly segmented into 5-second windows, where they extracted 22 univariate features, such as: Auto-regressive (AR) modelling predictive error, Decorrelation time, Energy, Hjörth parameters mobility and complexity, Spectral edge frequency and power, mean, variance, skewness and kurtosis, and Energy of wavelet coefficients. This output resulted in a total of 132 features from the combination of six scalp EEG input channels. We used the extracted features to train the linear-SVM classification model, which is computationally light while presenting comparable performances in relatively high-dimensional spaces[3].

The training phase used a cross-fold validation method to get the required configuration parameters. They used a grid-search to optimise the hyperparameter C (known as cost and represents the trade-off between the classification margin and non-separable patterns). For the grid-search, they used 3-fold cross-validation with the first three chronological seizures: fold 1 used seizures #1 and #2 for training, and #3 for validation; fold 2 used seizures #1 and #3 for training, and #2 for validation; and fold 3 used seizures #2 and #3 for training, and #1 for validation. Over an array of possible values [2^1, 2^4, 2^7, 2^{10}, 2^{13}, 2^{16}]. A second optimisation step was performed around the best candidate C_0, using the array of values C_0*[$2^{-1.5}$, $2^{-0.5}$, $2^{0.5}$, $2^{1.5}$]. The remaining seizures comprised the testing group, we smoothed the classifier output with the Firing Power technique, with a threshold value of (0.5) to prevent rapid changes and, thus, be robust to noise.

We replaced the filtering/decomposition and feature extraction processes of the study from Direito et al.[3] with ours and added a filter feature selection method based on the ANOVA (Analysis of Variance) f-test, which estimates the degree of linear dependency between each feature and the target. We selected the most discriminative features according to their ranking. For the number of features, we used the first three chronological seizures for a grid-search (10, 20, 30, 40 features) that optimises the Sample Sensitivity (S_{ss}) and Sample Specificity (S_{sp}): $\sqrt{S_{ss} * S_{sp}}$. We also used this grid search to find the best pre-ictal period (10, 15, 20, 25, 30, 35, 40, 45, 50 minutes) and the cost C over an array of possible values [2^{-10}, 2^{-8}, 2^{-6}, 2^{-4}, 2^{-2}, 2^0, 2^2, 2^4, 2^6, 2^8, 2^{10}]. In testing, we also implemented the Firing Power to smooth the output over time, with the same threshold value of 0.5. We also used refractory periods as in Direito et al.[3]. This methodology is patient-specific.

Concept-Drifts adaptation algorithm details

In this section, we show the pseudocode for each proposed algorithm.

Algorithm 1 Backwards-Landmark Window Pseudocode illustrating the window adjustment method by optimising performance with SVMs.

```
input ← Z = S training samples in t batches of n samples (1-hour) each
for h ∈ 0, ..., t-1 do
  train SVM on samples [Z(t-h,1),...,Z(t,n)]
  compute the leave-one-out-estimate on samples [Z(t-h,1),...,Z(t,n)]
  m = estimate.linear_regression(last 12 hours).slope
  if m ≥ 0.05 then
    stop window adjustment
  end if
end for
output ← W = window size which minimises the leave-one-out-estimate
```

Algorithm 2 Seizure-batch Regression Pseudocode illustrating the concept drift tracker via regression.

input ← $Z = S$ training seizures
test seizure index ← T_i = length of Z
validation seizure index ← $V_i = T_i$ - 1 (last training seizure)
for $h \in 0, ..., V_i$ **do**
  combination of seizures ← C = S($h : V_i$)
  weight vector combination of seizures ← $\vec{w_1}$ = logistic regression(C)
  weight vector validation seizure ← $\vec{w_2}$ = logistic regression[$S(V_i)$]
  $\theta_h = \theta(\vec{w_1}, \vec{w_2})$
end for
output ← $C_{optimal}$ = combination with the smallest angle

Algorithm 3 Dynamic Weighted Ensemble Pseudocode illustrating the dynamic integration of classifiers for handling concept drift.

input ← $Z = S$ training seizures
window size ← W = 1-hour
for seizure index ∈ 0, ..., S-1 **do**
  windows = windowing(S[seizure index)]
  for $h \in 0, ..., t$-1 **do**
    segment = windows(h)
    classifier(seizure index,h).train[segment + S(seizure index).SOP]
    W(seizure index,h) = classifier(seizure index,h).accuracy[$S(t-1)$.last-2-hours]
  end for
end for
output ← W = ensemble weights

Results

Iterative Retraining

Table 2. Training parameters and performance obtained for each patient for the Control data partitioning.

Control partitioning					
**Patient**	**SOP**	**k**	**C**	$\mathbf{SS}_{sample}$	$\mathbf{SP}_{sample}$
402	10	20	2^{-10}	0.43	0.71
8902	20	40	2^{-10}	0.88	0.83
11002	15	10	2^{6}	0.45	0.71
16202	15	40	2^{-4}	0.64	0.83
21902	10	10	2^{-10}	0.67	0.61
23902	50	40	2^{-10}	0.68	0.53
26102	50	40	2^{8}	0.31	0.62
30802	50	30	2^{-10}	0.90	0.79
32702	15	10	2^{-10}	0.75	0.69
45402	15	40	2^{-10}	0.72	0.56
46702	40	40	2^{8}	0.22	0.67
50802	15	10	2^{-4}	0.83	0.84
53402	40	10	2^{-10}	0.48	0.66
55202	10	30	2^{-4}	0.53	0.72
56402	10	10	2^{-6}	0.74	0.68
58602	10	10	2^{0}	0.30	0.70
59102	15	10	2^{-10}	0.63	0.45
60002	15	10	2^{-4}	0.54	0.72
64702	35	20	2^{-10}	0.46	0.67
75202	30	30	2^{-8}	0.72	0.83
80702	45	30	2^{2}	0.41	0.73
85202	15	30	2^{2}	0.45	0.67
93402	50	10	2^{8}	0.52	0.54
93902	40	30	2^{-2}	0.62	0.56
94402	10	40	2^{2}	0.40	0.68
95202	10	10	2^{-10}	0.74	0.66
96002	40	10	2^{6}	0.84	0.64
98102	35	10	2^{8}	0.50	0.53
101702	10	10	2^{-8}	0.58	0.53
102202	50	10	2^{0}	0.35	0.66
104602	15	40	2^{8}	0.35	0.66
109502	10	10	2^{-10}	0.58	0.53
112802	10	30	2^{-10}	0.55	0.56
113902	45	30	2^{-8}	0.42	0.55
114702	35	20	2^{2}	0.28	0.70
114902	20	20	2^{0}	0.47	0.63
123902	10	40	2^{-6}	0.80	0.85
Overall	-	-	-	0.56 ± 0.18	0.66 ± 0.10

Table 3. Training parameters and performance obtained for each patient for the Add-One-Forget-One data partitioning and iterative retraining.

		**Add-One-Forget-One**			
**Patient**	**SOP**	**k**	**C**	$\mathbf{SS}_{sample}$	$\mathbf{SP}_{sample}$
402	40, 20	10, 10	$2^{-10}, 2^{-4}$	0.53, 0.42	0.62, 0.65
8902	20, 20	10, 10	$2^{-10}, 2^{-10}$	0.87, 0.91	0.84, 0.81
11002	15	20	2^{-10}	0.51	0.68
16202	15, 10, 50, 20	30, 30, 10, 40	$2^{-10}, 2^{-4}, 2^{-8}, 2^{-6}$	0.63, 0.42, 0.52, 0.24	0.82, 0.73, 0.79, 0.71
21902	10	20	2^{2}	0.66	0.61
23902	45, 10	40, 40	$2^{-10}, 2^{-10}$	0.69, 0.51	0.52, 0.66
26102	50	40	2^{6}	0.31	0.59
30802	50, 50, 35, 10, 50	40, 40, 30, 10, 10	$2^{-8}, 2^{-4}, 2^{-8}, 2^{-10}, 2^{-10}$	0.90, 0.58, 0.38, 0.56, 0.78	0.80, 0.79, 0.64, 0.71, 0.53
32702	15, 20	10, 20	$2^{-10}, 2^{-10}$	0.77, 0.75	0.67, 0.72
45402	15	10	2^{-2}	0.72	0.53
46702	45, 15	30, 10	$2^{-4}, 2^{-10}$	0.23, 0.65	0.67, 0.78
50802	15, 20	30, 20	$2^{-8}, 2^{-10}$	0.80, 0.58	0.83, 0.79
53402	40	30	2^{8}	0.47	0.65
55202	10, 15, 25, 10, 10	10, 10, 10, 10, 10	$2^{-10}, 2^{-10}, 2^{-10}, 2^{-10}, 2^{-8}$	0.61, 0.65, 0.70, 0.89, 0.63	0.66, 0.70, 0.49, 0.73, 0.58
56402	10	10	2^{-8}	0.80	0.64
58602	10, 10, 10	10, 40, 40	$2^{4}, 2^{-4}, 2^{-10}$	0.24, 0.33, 0.53	0.71, 0.79, 0.75
59102	15, 50	40, 40	$2^{4}, 2^{-2}$	0.64, 0.36	0.45, 0.44
60002	15, 30, 40	10, 10, 40	$2^{-8}, 2^{-8}, 2^{-10}$	0.58, 0.20, 0.55	0.72, 0.67, 0.7
64702	30, 20	30, 40	$2^{-10}, 2^{-10}$	0.42, 0.80	0.68, 0.67
75202	30, 10, 10, 50	30, 20, 40, 40	$2^{-10}, 2^{-10}, 2^{-10}, 2^{2}$	0.71, 0.70, 0.68, 0.83	0.83, 0.80, 0.70, 0.56
80702	45, 45, 10	20, 20, 40	$2^{-10}, 2^{-10}, 2^{-8}$	0.38, 0.54, 0.73	0.73, 0.58, 0.36
85202	15, 40	30, 20	$2^{6}, 2^{8}$	0.45, 0.63	0.66, 0.67
93402	50, 20	10, 10	$2^{-10}, 2^{2}$	0.57, 0.45	0.52, 0.75
93902	40, 50, 15	30, 30, 10	$2^{-10}, 2^{-10}, 2^{-10}$	0.60, 0.73, 0.58	0.57, 0.59, 0.54
94402	10, 40, 15, 15	10, 10, 30, 40	$2^{4}, 2^{-10}, 2^{-6}, 2^{-10}$	0.41, 0.23, 0.13, 0.54	0.63, 0.64, 0.77, 0.84
95202	10, 45, 50, 25	10, 40, 10, 10	$2^{-10}, 2^{8}, 2^{-10}, 2^{-10}$	0.76, 0.33, 0.22, 0.55	0.65, 0.71, 0.64, 0.63
96002	35, 20, 15, 30	40, 30, 40, 20	$2^{-10}, 2^{-2}, 2^{0}, 2^{-2}$	0.81, 0.45, 0.52, 0.63	0.65, 0.66, 0.72, 0.79
98102	35, 45	40, 20	$2^{8}, 2^{-2}$	0.51, 0.6	0.52, 0.68
101702	10, 45	20, 30	$2^{-6}, 2^{8}$	0.62, 0.47	0.50, 0.60
102202	45, 50, 45, 10	30, 40, 30, 20	$2^{0}, 2^{2}, 2^{-2}, 2^{-6}$	0.33, 0.43, 0.31, 0.24	0.65, 0.51, 0.53, 0.57
104602	25, 50	10, 10	$2^{-10}, 2^{-10}$	0.43, 0.60	0.62, 0.67
109502	10	10	2^{-6}	0.57	0.53
112802	10, 10, 10	10, 30, 30	$2^{-10}, 2^{-4}, 2^{8}$	0.54, 0.26, 0.33	0.58, 0.63, 0.64
113902	45, 15, 45	20, 30, 40	$2^{-10}, 2^{-10}, 2^{-10}$	0.42, 0.64, 0.46	0.56, 0.73, 0.61
114702	35, 15, 10, 50, 50	30, 40, 20, 10, 40	$2^{-8}, 2^{6}, 2^{6}, 2^{0}, 2^{-6}$	0.26, 0.24, 0.18, 0.64, 0.62	0.73, 0.77, 0.85, 0.54, 0.63
114902	20, 35, 30, 35	10, 30, 10, 40	$2^{-10}, 2^{6}, 2^{-10}, 2^{-10}$	0.52, 0.15, 0.3, 0.43	0.62, 0.66, 0.76, 0.76
123902	10, 10	40, 10	$2^{-6}, 2^{-4}$	0.79, 0.65	0.84, 0.83
Overall	-	-	-	0.53 ± 0.19	0.66 ± 0.10

Table 4. Training parameters and performance obtained for each patient for the Chronological Accumulation data partitioning and iterative retraining.

			Chronological Accumulation		
**Patient**	**SOP**	**k**	**C**	$\mathbf{SS}_{sample}$	$\mathbf{SP}_{sample}$
402	40, 50	10, 40	$2^{-10}, 2^{6}$	0.54, 0.59	0.62, 0.59
8902	20, 20	10, 10	$2^{0}, 2^{4}$	0.87, 0.91	0.84, 0.85
11002	15	20	2^{-10}	0.50	0.69
16202	15, 10, 15, 10	10, 30, 30, 40	$2^{-10}, 2^{2}, 2^{2}, 2^{6}$	0.59, 0.49, 0.59, 0.47	0.83, 0.82, 0.74, 0.74
21902	10	40	2^{-10}	0.67	0.61
23902	45, 15	40, 10	$2^{-8}, 2^{-10}$	0.69, 0.48	0.52, 0.68
26102	50	40	2^{6}	0.31	0.58
30802	50, 50, 50, 35, 35	40, 20, 20, 40, 10	$2^{-8}, 2^{-10}, 2^{-10}, 2^{-10}, 2^{-10}$	0.90, 0.72, 0.76, 0.79, 0.79	0.79, 0.78, 0.65, 0.66, 0.59
32702	15, 15	10, 10	$2^{-2}, 2^{-10}$	0.74, 0.82	0.71, 0.71
45402	10	40	2^{-10}	0.66	0.60
46702	35, 25	40, 40	$2^{-2}, 2^{8}$	0.20, 0.49	0.68, 0.63
50802	15, 20	30, 30	$2^{-10}, 2^{-10}$	0.80, 0.73	0.84, 0.77
53402	45	20	2^{-4}	0.48	0.67
55202	10, 10, 10, 10, 10	10, 10, 10, 10, 10	$2^{-10}, 2^{-10}, 2^{-10}, 2^{-10}, 2^{-8}$	0.50, 0.74, 0.69, 0.67, 0.71	0.73, 0.67, 0.55, 0.64, 0.61
56402	10	20	2^{-10}	0.79	0.64
58602	10, 10, 15	40, 40, 30	$2^{-10}, 2^{-10}, 2^{-10}$	0.26, 0.62, 0.56	0.72, 0.67, 0.62
59102	15, 50	10, 10	$2^{-10}, 2^{-10}$	0.66, 0.52	0.44, 0.43
60002	15, 20, 15	30, 20, 20	$2^{-10}, 2^{-10}, 2^{-8}$	0.55, 0.41, 0.36	0.71, 0.69, 0.63
64702	25, 15	30, 40	$2^{-6}, 2^{-4}$	0.41, 0.58	0.68, 0.68
75202	30, 10, 30, 40	10, 10, 10, 30	$2^{-4}, 2^{-10}, 2^{-10}, 2^{-4}$	0.71, 0.73, 0.69, 0.68	0.83, 0.82, 0.62, 0.61
80702	45, 45, 40	40, 40, 40	$2^{8}, 2^{4}, 2^{0}$	0.43, 0.54, 0.49	0.73, 0.69, 0.69
85202	15, 15	20, 30	$2^{0}, 2^{2}$	0.44, 0.57	0.66, 0.69
93402	50, 20	20, 40	$2^{4}, 2^{-6}$	0.57, 0.44	0.52, 0.75
93902	40, 45, 40	40, 10, 30	$2^{0}, 2^{-6}, 2^{-6}$	0.60, 0.78, 0.73	0.57, 0.53, 0.44
94402	20, 20, 50, 10	10, 10, 30, 10	$2^{0}, 2^{-4}, 2^{0}, 2^{-8}$	0.46, 0.23, 0.28, 0.27	0.57, 0.69, 0.58, 0.65
95202	10, 30, 10, 40	10, 30, 40, 10	$2^{-10}, 2^{4}, 2^{0}, 2^{-10}$	0.74, 0.28, 0.68, 0.55	0.66, 0.62, 0.66, 0.55
96002	30, 15, 25, 25	40, 40, 40, 30	$2^{-2}, 2^{4}, 2^{-10}, 2^{-10}$	0.82, 0.56, 0.62, 0.62	0.64, 0.66, 0.64, 0.65
98102	35, 50	40, 10	$2^{-8}, 2^{2}$	0.50, 0.69	0.52, 0.65
101702	10, 40	30, 40	$2^{-4}, 2^{-2}$	0.49, 0.60	0.60, 0.51
102202	45, 50, 20, 50	20, 10, 40, 40	$2^{-4}, 2^{-4}, 2^{-2}, 2^{0}$	0.35, 0.46, 0.52, 0.51	0.65, 0.75, 0.67, 0.55
104602	35, 35	20, 20	$2^{-10}, 2^{-4}$	0.42, 0.48	0.61, 0.66
109502	10	10	2^{-10}	0.56	0.54
112802	45, 50, 10	10, 30, 40	$2^{2}, 2^{-6}, 2^{-4}$	0.31, 0.30, 0.51	0.70, 0.62, 0.44
113902	45, 10, 50	40, 30, 10	$2^{6}, 2^{-4}, 2^{8}$	0.43, 0.50, 0.65	0.55, 0.69, 0.47
114702	35, 45, 10, 15, 15	30, 40, 40, 10, 30	$2^{-10}, 2^{-4}, 2^{-10}, 2^{-10}, 2^{-10}$	0.25, 0.36, 0.44, 0.4, 0.57	0.73, 0.55, 0.63, 0.63, 0.53
114902	20, 20, 35, 35	20, 30, 10, 10	$2^{-4}, 2^{2}, 2^{-10}, 2^{-10}$	0.46, 0.37, 0.54, 0.6	0.65, 0.59, 0.56, 0.61
123902	15, 10	40, 10	$2^{-10}, 2^{-10}$	0.80, 0.64	0.81, 0.85
Overall	-	-	-	0.56 ± 0.16	0.64 ± 0.10

Table 5. Testing performance obtained for each patient with the Control data partitioning method.

Control partitioning

Patient	Evaluated seizures	SOP	SS	FPR/h	SS Surrogate	p-value	Above chance
402	2	10	0.50	5.34	0.17 ± 0.24	0.00	•
8902	2	20	1.00	0.16	0.10 ± 0.20	0.00	•
11002	1	15	1.00	0.57	0.07 ± 0.25	0.00	•
16202	4	15	0.00	0.07	0.03 ± 0.08	0.96	
21902	1	10	0.00	1.42	0.10 ± 0.30	0.96	
23902	2	50	0.00	2.93	0.68 ± 0.30	1.00	
26102	1	50	1.00	1.49	0.53 ± 0.50	0.00	•
30802	5	50	0.60	0.43	0.37 ± 0.16	0.00	•
32702	2	15	0.00	0.45	0.07 ± 0.17	0.98	
45402	1	15	0.00	3.40	0.33 ± 0.47	1.00	
46702	2	40	0.50	1.77	0.38 ± 0.36	0.04	•
50802	2	15	0.00	0.47	0.07 ± 0.17	0.98	
53402	1	40	0.00	0.99	0.30 ± 0.46	1.00	
55202	5	10	0.40	2.32	0.28 ± 0.20	0.00	•
56402	1	10	1.00	5.94	0.33 ± 0.47	0.00	•
58602	3	10	0.00	2.54	0.19 ± 0.28	1.00	
59102	2	15	0.50	10.04	0.48 ± 0.42	0.42	
60002	3	15	0.00	1.03	0.16 ± 0.22	1.00	
64702	2	35	0.50	0.89	0.15 ± 0.23	0.00	•
75202	4	30	0.00	0.11	0.03 ± 0.08	0.98	
80702	3	45	0.67	1.74	0.44 ± 0.22	0.00	•
85202	2	15	0.00	0.17	0.02 ± 0.09	0.84	
93402	2	50	0.50	3.89	0.85 ± 0.29	1.00	
93902	3	40	0.33	0.46	0.29 ± 0.29	0.21	
94402	4	10	0.00	3.14	0.23 ± 0.23	1.00	
95202	4	10	0.25	1.10	0.13 ± 0.14	0.00	•
96002	4	40	0.25	2.00	0.58 ± 0.28	1.00	
98102	2	35	0.50	0.21	0.10 ± 0.20	0.00	•
101702	2	10	0.50	2.39	0.25 ± 0.25	0.00	•
102202	4	50	0.50	0.32	0.13 ± 0.12	0.00	•
104602	2	15	0.50	0.88	0.23 ± 0.31	0.00	•
109502	1	10	1.00	3.23	0.33 ± 0.47	0.00	•
112802	3	10	0.33	4.49	0.38 ± 0.25	0.82	
113902	3	45	0.67	2.69	0.54 ± 0.24	0.00	•
114702	5	35	0.00	0.25	0.18 ± 0.17	1.00	
114902	4	20	0.00	0.13	0.01 ± 0.04	0.84	
123902	2	10	0.00	0.00	0.00 ± 0.00	-	
Overall	-	-	0.35 ± 0.35	1.88 ± 2.05	0.25 ± 0.50	-	17

Table 6. Testing parameters and performance obtained for each patient for with the Add-One-Forget-One data partitioning and iterative retraining method.

		**Add-One-Forget-One**					
**Patient**	**Evaluated seizures**	**SOP**	**SS**	**FPR/h**	**SS Surrogate**	**p-value**	**Above chance**
402	2	40, 20	0.00	1.33	0.15 ± 0.23	1.00	
8902	2	20, 20	0.00	0.30	0.10 ± 0.20	0.99	
11002	1	15	0.00	4.53	0.37 ± 0.48	1.00	
16202	4	15, 10, 50, 20	0.75	0.34	0.11 ± 0.14	0.00	•
21902	1	10	1.00	16.19	0.40 ± 0.49	0.00	•
23902	2	45, 10	1.00	2.22	0.25 ± 0.25	0.00	•
26102	1	50	1.00	0.40	0.23 ± 0.42	0.00	•
30802	5	50, 50, 35, 10, 50	0.40	0.81	0.30 ± 0.17	0.00	•
32702	2	15, 20	1.00	1.69	0.20 ± 0.28	0.00	•
45402	1	15	1.00	3.63	0.33 ± 0.47	0.00	•
46702	2	45, 15	1.00	0.57	0.22 ± 0.31	0.00	•
50802	2	15, 20	1.00	0.59	0.12 ± 0.21	0.00	•
53402	1	40	1.00	0.84	0.20 ± 0.41	0.00	•
55202	5	10, 15, 25, 10, 10	0.60	1.38	0.21 ± 0.19	0.00	•
56402	1	10	1.00	4.57	0.27 ± 0.44	0.00	•
58602	3	10, 10, 10	0.67	1.68	0.14 ± 0.19	0.00	•
59102	2	15, 50	1.00	2.08	0.55 ± 0.30	0.00	•
60002	3	15, 30, 40	0.67	0.91	0.21 ± 0.20	0.00	•
64702	2	30, 20	0.50	1.89	0.32 ± 0.27	0.00	•
75202	4	30, 10, 10, 50	0.50	0.50	0.32 ± 0.18	0.00	•
80702	3	45, 45, 10	0.67	1.17	0.31 ± 0.27	0.00	•
85202	2	15, 40	0.00	1.04	0.37 ± 0.26	1.00	
93402	2	50, 20	0.50	0.45	0.22 ± 0.28	0.00	•
93902	3	40, 50, 15	0.67	2.21	0.32 ± 0.27	0.00	•
94402	4	10, 40, 15, 15	0.50	0.73	0.15 ± 0.14	0.00	•
95202	4	10, 45, 50, 25	0.75	0.53	0.16 ± 0.16	0.00	•
96002	4	35, 20, 15, 30	0.50	1.15	0.23 ± 0.22	0.00	•
98102	2	35, 45	0.50	0.54	0.30 ± 0.33	0.00	•
101702	2	10, 45	0.50	1.60	0.30 ± 0.28	0.00	•
102202	4	45, 50, 45, 10	0.50	0.42	0.15 ± 0.19	0.00	•
104602	2	25, 50	1.00	0.75	0.32 ± 0.33	0.00	•
109502	1	10	0.00	0.00	0.00 ± 0.00	-	
112802	3	10, 10, 10	0.67	3.06	0.22 ± 0.22	0.00	•
113902	3	45, 15, 45	0.67	1.20	0.32 ± 0.27	0.00	•
114702	5	35, 15, 10, 50, 50	1.00	0.99	0.31 ± 0.22	0.00	•
114902	4	20, 35, 30, 35	0.75	0.62	0.23 ± 0.23	0.00	•
123902	2	10, 10	0.00	0.64	0.05 ± 0.15	0.96	
Overall	-	-	0.63 ± 0.34	1.72 ± 2.65	0.26 ± 0.51	-	31

Table 7. Testing parameters and performance obtained for each patient for with the Chronological Accumulation data partitioning and iterative retraining method.

Chronological Accumulation

Patient	Evaluated seizures	SOP	SS	FPR/h	SS Surrogate	p-value	Above chance
402	2	40, 50	0.50	0.00	0.00 ± 0.00	0.00	•
8902	2	20, 20	0.50	0.25	0.17 ± 0.24	0.00	•
11002	1	15	0.00	3.64	0.30 ± 0.46	1.00	
16202	4	15, 10, 15, 10	1.00	0.33	0.07 ± 0.13	0.00	•
21902	1	10	1.00	12.19	0.37 ± 0.48	0.00	•
23902	2	45, 15	0.50	1.95	0.38 ± 0.33	0.03	•
26102	1	50	1.00	0.31	0.13 ± 0.34	0.00	•
30802	5	50, 50, 50, 35, 35	0.60	0.70	0.35 ± 0.09	0.00	•
32702	2	15, 15	0.50	1.74	0.20 ± 0.28	0.00	•
45402	1	10	0.00	2.08	0.27 ± 0.44	1.00	
46702	2	35, 25	1.00	0.47	0.20 ± 0.24	0.00	•
50802	2	15, 20	1.00	0.61	0.02 ± 0.09	0.00	•
53402	1	45	1.00	0.89	0.23 ± 0.42	0.00	•
55202	5	10, 10, 10, 10, 10	0.40	1.99	0.25 ± 0.18	0.00	•
56402	1	10	1.00	4.53	0.30 ± 0.46	0.00	•
58602	3	10, 10, 15	0.33	1.87	0.20 ± 0.25	0.00	•
59102	2	15, 50	1.00	2.27	0.47 ± 0.22	0.00	•
60002	3	15, 20, 15	0.67	1.27	0.20 ± 0.2	0.00	•
64702	2	25, 15	1.00	2.26	0.35 ± 0.32	0.00	•
75202	4	30, 10, 30, 40	1.00	0.34	0.17 ± 0.19	0.00	•
80702	3	45, 45, 40	0.67	0.41	0.12 ± 0.16	0.00	•
85202	2	15, 15	0.50	0.38	0.03 ± 0.12	0.00	•
93402	2	50, 20	0.50	0.77	0.30 ± 0.28	0.00	•
93902	3	40, 45, 40	1.00	2.04	0.37 ± 0.29	0.00	•
94402	4	20, 20, 50, 10	0.50	0.50	0.10 ± 0.14	0.00	•
95202	4	10, 30, 10, 40	1.00	0.47	0.16 ± 0.15	0.00	•
96002	4	30, 15, 25, 25	0.50	2.49	0.36 ± 0.24	0.00	•
98102	2	35, 50	1.00	0.44	0.18 ± 0.27	0.00	•
101702	2	10, 40	0.50	1.24	0.25 ± 0.25	0.00	•
102202	4	45, 50, 20, 50	0.25	0.17	0.03 ± 0.08	0.00	•
104602	2	35, 35	1.00	0.57	0.25 ± 0.25	0.00	•
109502	1	10	0.00	0.00	0.00 ± 0.00	-	
112802	3	45, 50, 10	1.00	0.21	0.21 ± 0.20	0.00	•
113902	3	45, 10, 50	0.67	1.67	0.48 ± 0.29	0.00	•
114702	5	35, 45, 10, 15, 15	1.00	1.36	0.19 ± 0.13	0.00	•
114902	4	20, 20, 35, 35	0.75	0.34	0.11 ± 0.15	0.00	•
123902	2	15, 10	0.50	0.63	0.12 ± 0.21	0.00	•
Overall	-	-	0.68 ± 0.32	1.44 ± 2.06	0.23 ± 0.48	-	34

Table 8. Training parameters and performance obtained for each patient with the Backwards-Landmark Window and the Add-One-Forget-One data partitioning and iterative retraining method.

**Add-One-Forget-One - Backwards-Landmark Window**					
**Patient**	**SOP**	**k**	**C**	**SS**$_{sample}$	**SP**$_{sample}$
402	40, 40	40, 40	2^{-2},2^{-4}	0.50, 0.07	0.84, 0.53
8902	20, 30	10, 10	2^{-10},2^{0}	0.82, 0.97	0.78, 0.78
11002	20	10	2^{-4}	0.53	0.72
16202	15, 25, 50, 10	40, 30, 40, 20	2^{-10},2^{-6},2^{-2},2^{-10}	0.47, 0.70, 0.25, 0.36	0.82, 0.59, 0.75, 0.74
21902	40	10	2^{6}	0.71	0.73
23902	50, 10	20, 40	2^{-2},2^{-6}	0.81, 0.44	0.56, 0.75
26102	50	20	2^{-10}	0.47	0.72
30802	10, 10, 50, 25, 50	40, 10, 40, 20, 40	2^{-10},2^{-10},2^{2},2^{-10},2^{-10}	0.59, 0.26, 0.45, 0.59, 0.65	0.84, 0.86, 0.53, 0.70, 0.63
32702	15, 15	20, 10	2^{-10},2^{-2}	0.82, 0.83	0.76, 0.75
45402	50	20	2^{2}	0.67	0.57
46702	30, 10	20, 10	2^{-8},2^{-10}	0.37, 0.51	0.76, 0.85
50802	15, 25	10, 20	2^{-4},2^{-10}	0.73, 0.62	0.84, 0.87
53402	45	40	2^{0}	0.52	0.66
55202	35, 10, 25, 10, 10	10, 20, 30, 10, 30	2^{-6},2^{-10},2^{2},2^{-10},2^{-6}	0.62, 0.51, 0.60, 0.94, 0.53	0.53, 0.72, 0.56, 0.67, 0.69
56402	15	10	2^{-4}	0.80	0.67
58602	10, 10, 20	10, 20, 30	2^{-10},2^{-10},2^{-10}	0.45, 0.57, 0.50	0.79, 0.85, 0.73
59102	20, 15	20, 40	2^{-10},2^{-4}	0.48, 0.48	0.59, 0.37
60002	20, 15, 35	20, 20, 40	2^{-6},2^{-8},2^{2}	0.46, 0.51, 0.58	0.80, 0.66, 0.54
64702	50, 15	20, 10	2^{-10},2^{-4}	0.37, 0.54	0.56, 0.62
75202	30, 10, 45, 35	10, 10, 10, 30	2^{-4},2^{-10},2^{-10},2^{-4}	0.68, 0.53, 0.31, 0.56	0.85, 0.83, 0.85, 0.49
80702	10, 50, 50	40, 40, 40	2^{8},2^{4},2^{0}	0.66, 0.54, 0.54	0.34, 0.47, 0.54
85202	10, 30	20, 30	2^{0},2^{2}	0.21, 0.59	0.85, 0.55
93402	15, 20	20, 40	2^{4},2^{-6}	0.45, 0.67	0.71, 0.71
93902	30, 45, 20	40, 10, 30	2^{0},2^{-6},2^{-6}	0.42, 0.41, 0.75	0.34, 0.24, 0.76
94402	10, 20, 35, 15	10, 10, 30, 10	2^{0},2^{-4},2^{0},2^{-8}	0.51, 0.17, 0.39, 0.46	0.52, 0.79, 0.82, 0.99
95202	10, 35, 40, 10	10, 30, 40, 10	2^{-10},2^{4},2^{0},2^{-10}	0.82, 0.35, 0.39, 0.76	0.57, 0.66, 0.68, 0.90
96002	40, 20, 15, 50	40, 40, 40, 30	2^{-2},2^{4},2^{-10},2^{-10}	0.43, 0.23, 0.85, 0.33	0.63, 0.83, 0.66, 0.87
98102	25, 10	40, 10	2^{-8},2^{2}	0.22, 0.71	0.57, 0.68
101702	30, 50	30, 40	2^{-4},2^{-2}	0.63, 0.70	0.51, 0.58
102202	10, 20, 10, 15	20, 10, 40, 40	2^{-4},2^{-4},2^{-2},2^{0}	0.54, 0.49, 0.22, 0.56	0.63, 0.7, 0.9, 0.54
104602	30, 40	20, 20	2^{-10},2^{-4}	0.76, 0.79	0.55, 0.66
109502	10	10	2^{-10}	0.37	0.79
112802	10, 40, 25	10, 30, 40	2^{2},2^{-6},2^{-4}	0.43, 0.35, 0.51	0.56, 0.73, 0.41
113902	50, 10, 50	40, 30, 10	2^{6},2^{-4},2^{8}	0.44, 0.72, 0.73	0.52, 0.71, 0.56
114702	35, 15, 15, 50, 15	30, 40, 40, 10, 30	2^{-10},2^{-4},2^{-10},2^{-10},2^{-10}	0.22, 0.17, 0.67, 0.41, 0.56	0.76, 0.93, 0.7, 0.53, 0.74
114902	25, 50, 15, 20	20, 30, 10, 10	2^{-4},2^{2},2^{-10},2^{-10}	0.67, 0.05, 0.25, 0.75	0.44, 0.81, 0.93, 0.73
123902	40, 10	40, 10	2^{-10},2^{-10}	0.80, 0.33	0.77, 0.9
Overall	-	-	-	0.53 ± 0.19	0.70 ± 0.15

Table 9. Training parameters and performance obtained for each patient with the Seizure-batch Regression and the Add-One-Forget-One data partitioning and iterative retraining method.

**Add-One-Forget-One - Seizure-batch Regression**					
**Patient**	**SOP**	**k**	**C**	$\mathbf{SS}_{sample}$	$\mathbf{SP}_{sample}$
402	10, 10	10, 10	$2^{-10},2^{-4}$	0.52, 0.25	0.78, 0.53
8902	25, 30	10, 10	$2^{-10},2^{-10}$	0.77, 0.91	0.76, 0.85
11002	10	20	2^{-10}	0.42	0.71
16202	40, 10, 15, 30	30, 30, 10, 40	$2^{-10},2^{-4},2^{-8},2^{-6}$	0.65, 0.22, 0.15, 0.14	0.76, 0.85, 0.81, 0.81
21902	50	20	2^{2}	0.68	0.7
23902	50, 10	40, 40	$2^{-10},2^{-10}$	0.37, 0.55	0.62, 0.67
26102	50	40	2^{6}	0.26	0.74
30802	35, 15, 20, 50, 50	40, 40, 30, 10, 10	$2^{-8},2^{-4},2^{-8},2^{-10},2^{-10}$	0.66, 0.31, 0.56, 0.54, 0.65	0.85, 0.9, 0.56, 0.77, 0.64
32702	15, 25	10, 20	$2^{-10},2^{-10}$	0.57, 0.61	0.74, 0.71
45402	15	10	2^{-2}	0.36	0.76
46702	40, 10	30, 10	$2^{-4},2^{-10}$	05, 0.44	0.87, 0.82
50802	15, 25	30, 20	$2^{-8},2^{-10}$	0.7, 0.39	0.86, 0.9
53402	30	30	2^{8}	0.26	0.76
55202	10, 10, 10, 10, 50	10, 10, 10, 10, 10	$2^{-10},2^{-10},2^{-10},2^{-10},2^{-8}$	0.73, 0.49, 0.52, 0.62, 0.55	0.55, 0.9, 0.6, 0.6, 0.51
56402	25	10	2^{-8}	0.41	0.74
58602	10, 10, 20	10, 40, 40	$2^{4},2^{-4},2^{-10}$	0.16, 0.2, 0.36	0.92, 0.95, 0.8
59102	50, 15	40, 40	$2^{4},2^{-2}$	0.38, 0.54	0.42, 0.32
60002	20, 20, 40	10, 10, 40	$2^{-8},2^{-8},2^{-10}$	0.44, 0.26, 0.44	0.77, 0.77, 0.83
64702	40, 25	30, 40	$2^{-10},2^{-10}$	0.44, 0.43	0.67, 0.79
75202	30, 10, 50, 50	30, 20, 30, 10	$2^{-10},2^{-10},2^{-10},2^{-8}$	0.74, 0.6, 0.51, 0.84	0.83, 0.87, 0.83, 0.43
80702	35, 50, 15	40, 40, 30	$2^{-10},2^{-8},2^{-10}$	0.3, 0.63, 0.53	0.79, 0.55, 0.51
85202	10, 15	20, 10	$2^{-10},2^{-10}$	0.26, 0.5	0.85, 0.63
93402	20, 15	10, 10	$2^{0},2^{8}$	0.4, 0.46	0.66, 0.53
93902	50, 50, 15	10, 20, 20	$2^{-10},2^{-10},2^{-8}$	0.56, 0.45, 0.43	0.62, 0.84, 0.75
94402	10, 35, 30, 10	10, 20, 20, 30	$2^{8},2^{-8},2^{8},2^{-2}$	0.42, 0.5, 0.36, 0.27	0.56, 0.55, 0.69, 0.95
95202	10, 40, 45, 25	10, 40, 30, 30	$2^{-10},2^{8},2^{6},2^{-10}$	0.51, 0.36, 0.33, 0.66	0.65, 0.67, 0.56, 0.67
96002	25, 40, 35, 45	20, 30, 40, 20	$2^{-2},2^{-6},2^{-6},2^{8}$	0.52, 0.29, 0.61, 0.39	0.7, 0.63, 0.73, 0.86
98102	25, 35	20, 30	$2^{-4},2^{-2}$	0.31, 0.53	0.57, 0.7
101702	35, 50	20, 40	$2^{-2},2^{-8}$	0.4, 0.29	0.62, 0.77
102202	50, 45, 50, 50	30, 40, 30, 10	$2^{-6},2^{-4},2^{-2},2^{-6}$	06, 0.32, 0.27, 09	0.97, 0.48, 0.48, 0.75
104602	25, 45	10, 30	$2^{-8},2^{-10}$	0.38, 0.75	0.68, 0.67
109502	20	10	2^{-10}	0.35	0.57
112802	10, 15, 15	10, 10, 40	$2^{-10},2^{-6},2^{-10}$	0.65, 0.38, 0.19	0.49, 0.58, 0.79
113902	50, 10, 40	20, 40, 20	$2^{-10},2^{-6},2^{-2}$	0.13, 0.29, 0.42	0.77, 0.89, 0.66
114702	40, 30, 15, 45, 50	20, 20, 40, 10, 40	$2^{8},2^{-8},2^{-8},2^{-10},2^{-10}$	0.27, 04, 0.28, 0.52, 0.75	0.62, 0.84, 0.86, 0.55, 0.63
114902	25, 50, 50, 20	20, 30, 10, 10	$2^{0},2^{8},2^{-10},2^{-10}$	0.53, 04, 0.15, 0.67	0.44, 0.77, 0.91, 0.84
123902	50, 35	10, 20	$2^{-8},2^{-10}$	0.57, 0.2	0.83, 0.9
Overall	-	-	-	0.43 ± 0.20	0.71 ± 0.14

Table 10. Training parameters and performance obtained for each patient with the Dynamic Weighted Ensemble and the Add-One-Forget-One data partitioning and iterative retraining method.

**Add-One-Forget-One - Dynamic Weighted Ensemble**					
**Patient**	**SOP**	**k**	**C**	$\mathbf{SS}_{sample}$	$\mathbf{SP}_{sample}$
402	10, 15	10, 10	$2^{-10}, 2^{8}$	0.69, 0.19	0.65, 0.59
8902	15, 20	10, 10	$2^{0}, 2^{6}$	0.90, 0.97	0.68, 0.78
11002	10	20	2^{-10}	0.53	0.61
16202	45, 15, 10, 30	10, 40, 20, 40	$2^{-10}, 2^{8}, 2^{8}, 2^{6}$	0.83, 0.55, 0.46, 0.21	0.61, 0.59, 0.69, 0.71
21902	40	40	2^{-10}	0.84	0.58
23902	50, 10	40, 30	$2^{-8}, 2^{0}$	0.50, 0.63	0.53, 0.53
26102	50	40	2^{6}	0.66	0.35
30802	35, 15, 20, 25, 50	40, 10, 20, 30, 10	$2^{-8}, 2^{-10}, 2^{0}, 2^{-10}, 2^{-10}$	0.92, 0.40, 0.53, 0.59, 0.74	0.78, 0.85, 0.56, 0.73, 0.46
32702	15, 20	10, 20	$2^{-2}, 2^{-10}$	0.67, 0.86	0.70, 0.57
45402	50	40	2^{-10}	0.67	0.55
46702	30, 10	40, 10	$2^{-2}, 2^{4}$	0.37, 0.66	0.69, 0.75
50802	15, 20	30, 40	$2^{-10}, 2^{-10}$	0.82, 0.75	0.70, 0.8
53402	15	20	2^{-4}	0.22	0.72
55202	10, 10, 10, 10, 50	10, 10, 10, 20, 10	$2^{-10}, 2^{-10}, 2^{-6}, 2^{-2}, 2^{-10}$	0.84, 0.63, 0.67, 0.71, 0.62	0.52, 0.83, 0.56, 0.56, 0.45
56402	25	20	2^{-10}	0.46	0.72
58602	10, 10, 20	40, 40, 40	$2^{-10}, 2^{-10}, 2^{-8}$	0.15, 0.17, 0.65	0.78, 0.87, 0.73
59102	50, 50	10, 10	$2^{-10}, 2^{-10}$	0.41, 0.73	0.42, 0.14
60002	25, 25, 50	30, 10, 40	$2^{-10}, 2^{-10}, 2^{-10}$	0.56, 0.40, 0.68	0.65, 0.67, 0.69
64702	50, 25	30, 30	$2^{-6}, 2^{-6}$	0.64, 0.71	0.46, 0.55
75202	30, 10, 10, 10	30, 20, 40, 40	$2^{-10}, 2^{-10}, 2^{-10}, 2^{2}$	0.82, 0.70, 0.84, 0.57	0.75, 0.81, 0.75, 0.57
80702	30, 30, 45	20, 20, 40	$2^{-10}, 2^{-10}, 2^{-8}$	0.51, 0.67, 0.61	0.63, 0.40, 0.51
85202	10, 15	30, 20	$2^{6}, 2^{8}$	0.25, 0.65	0.79, 0.53
93402	20, 15	10, 10	$2^{-10}, 2^{2}$	0.64, 0.56	0.43, 0.37
93902	50, 50, 40	30, 30, 10	$2^{-10}, 2^{-10}, 2^{-10}$	0.71, 0.69, 0.43	0.51, 0.72, 0.63
94402	10, 50, 25, 10	10, 10, 30, 40	$2^{4}, 2^{-10}, 2^{-6}, 2^{-10}$	0.48, 0.82, 0.29, 0.49	0.62, 0.36, 0.71, 0.86
95202	10, 40, 35, 25	10, 40, 10, 10	$2^{-10}, 2^{8}, 2^{-10}, 2^{-10}$	0.77, 0.48, 0.42, 0.74	0.43, 0.57, 0.50, 0.59
96002	10, 40, 50, 45	40, 30, 40, 20	$2^{-10}, 2^{-2}, 2^{0}, 2^{-2}$	0.84, 0.55, 0.92, 0.55	0.56, 0.45, 0.64, 0.77
98102	25, 50	40, 20	$2^{8}, 2^{-2}$	0.58, 0.63	0.32, 0.58
101702	10, 50	20, 30	$2^{-6}, 2^{8}$	0.50, 0.40	0.57, 0.67
102202	45, 25, 50, 50	30, 40, 30, 20	$2^{0}, 2^{2}, 2^{-2}, 2^{-6}$	0.12, 0.17, 0.4, 0.11	0.88, 0.66, 0.28, 0.73
104602	20, 30	10, 10	$2^{-10}, 2^{-10}$	0.51, 0.80	0.58, 0.60
109502	30	10	2^{-6}	0.34	0.71
112802	10, 10, 30	10, 30, 30	$2^{-10}, 2^{-4}, 2^{8}$	0.77, 0.36, 0.26	0.37, 0.56, 0.71
113902	15, 15, 10	20, 30, 40	$2^{-10}, 2^{-10}, 2^{-10}$	0.35, 0.60, 0.59	0.65, 0.76, 0.64
114702	50, 30, 10, 45, 50	30, 40, 20, 10, 40	$2^{-8}, 2^{6}, 2^{6}, 2^{0}, 2^{-6}$	0.46, 0.17, 0.24, 0.6, 0.84	0.41, 0.66, 0.87, 0.51, 0.51
114902	25, 45, 35, 35	10, 30, 10, 40	$2^{-10}, 2^{6}, 2^{-10}, 2^{-10}$	0.70, 03, 0.25, 0.85	0.23, 0.76, 0.89, 0.58
123902	10, 20	40, 10	$2^{-6}, 2^{-4}$	0.92, 0.31	0.80, 0.88
Overall	-	-	-	0.56 ± 0.22	0.62 ± 0.15

Table 11. Testing parameters and performance obtained for each patient with the Backwards-Landmark Window and the Add-One-Forget-One data partitioning and iterative retraining method.

Add-One-Forget-One - Backwards-Landmark Window

Patient	Evaluated seizures	SOP	SS	FPR/h	SS Surrogate	p-value	Above chance
402	2	40, 40	1.00	0.00	0.00 ± 0.00	0.00	•
8902	2	20, 30	0.00	0.19	0.10 ± 0.20	0.99	
11002	1	20	1.00	0.00	0.00 ± 0.00	0.00	•
16202	4	15, 25, 50, 10	1.00	1.82	0.28 ± 0.15	0.00	•
21902	1	40	1.00	1.23	0.33 ± 0.47	0.00	•
23902	2	50, 10	1.00	2.99	0.32 ± 0.35	0.00	•
26102	1	50	1.00	2.88	0.53 ± 0.50	0.00	•
30802	5	10, 10, 50, 25, 50	0.60	0.79	0.10 ± 0.14	0.00	•
32702	2	15, 15	1.00	1.90	0.22 ± 0.31	0.00	•
45402	1	50	1.00	0.23	0.17 ± 0.37	0.00	•
46702	2	30, 10	0.50	1.46	0.23 ± 0.25	0.00	•
50802	2	15, 25	1.00	0.96	0.15 ± 0.26	0.00	•
53402	1	45	1.00	0.03	0.03 ± 0.18	0.00	•
55202	5	35, 10, 25, 10, 10	0.80	0.34	0.11 ± 0.13	0.00	•
56402	1	15	0.00	4.77	0.23 ± 0.42	1.00	
58602	3	10, 10, 20	0.67	0.73	0.16 ± 0.21	0.00	•
59102	2	20, 15	1.00	0.17	0.05 ± 0.15	0.00	•
60002	3	20, 15, 35	1.00	1.30	0.27 ± 0.22	0.00	•
64702	2	50, 15	0.50	1.49	0.37 ± 0.34	0.02	•
75202	4	30, 10, 45, 35	0.75	0.23	0.27 ± 0.14	0.00	•
80702	3	10, 50, 50	1.00	0.91	0.36 ± 0.23	0.00	•
85202	2	10, 30	0.00	1.61	0.22 ± 0.28	1.00	
93402	2	15, 20	0.50	0.11	0.03 ± 0.12	0.00	•
93902	3	30, 45, 20	1.00	1.42	0.38 ± 0.24	0.00	•
94402	4	10, 20, 35, 15	0.75	0.12	0.03 ± 0.08	0.00	•
95202	4	10, 35, 40, 10	0.75	1.83	0.35 ± 0.22	0.00	•
96002	4	40, 20, 15, 50	0.75	0.73	0.16 ± 0.16	0.00	•
98102	2	25, 10	1.00	2.37	0.40 ± 0.24	0.00	•
101702	2	30, 50	1.00	0.64	0.32 ± 0.27	0.00	•
102202	4	10, 20, 10, 15	0.25	0.36	0.06 ± 0.11	0.00	•
104602	2	30, 40	1.00	0.81	0.33 ± 0.27	0.00	•
109502	1	10	0.00	0.47	0.07 ± 0.25	0.92	
112802	3	10, 40, 25	1.00	0.83	0.18 ± 0.22	0.00	•
113902	3	50, 10, 50	0.67	1.13	0.37 ± 0.20	0.00	•
114702	5	35, 15, 15, 50, 15	0.60	0.74	0.19 ± 0.17	0.00	•
114902	4	25, 50, 15, 20	1.00	0.22	0.17 ± 0.21	0.00	•
123902	2	40, 10	0.50	0.17	0.03 ± 0.12	0.00	•
Overall	-	-	0.75 ± 0.33	1.03 ± 1.00	0.22 ± 0.47	-	33

Table 12. Testing parameters and performance obtained for each patient with the Seizure-batch Regression and the Add-One-Forget-One data partitioning and iterative retraining method.

Add-One-Forget-One - Seizure-batch Regression

Patient	Evaluated seizures	SOP	SS	FPR/h	SS Surrogate	p-value	Above chance
402	2	10, 10	0.00	5.07	0.17 ± 0.24	1.00	
8902	2	25, 30	0.50	0.31	0.13 ± 0.22	0.00	•
11002	1	10	0.00	11.96	0.47 ± 0.50	1.00	
16202	4	40, 10, 15, 30	0.25	0.61	0.13 ± 0.15	0.00	•
21902	1	50	1.00	0.19	0.13 ± 0.34	0.00	•
23902	2	50, 10	1.00	0.67	0.10 ± 0.24	0.00	•
26102	1	50	1.00	0.05	0.03 ± 0.18	0.00	•
30802	5	35, 15, 20, 50, 50	0.60	0.49	0.23 ± 0.17	0.00	•
32702	2	15, 25	1.00	1.81	0.30 ± 0.33	0.00	•
45402	1	15	1.00	97.86	0.60 ± 0.49	0.00	•
46702	2	40, 10	0.50	0.61	0.12 ± 0.25	0.00	•
50802	2	15, 25	1.00	0.41	0.03 ± 0.12	0.00	•
53402	1	30	1.00	0.03	0.00 ± 0.00	0.00	•
55202	5	10, 10, 10, 10, 50	0.60	1.20	0.23 ± 0.19	0.00	•
56402	1	25	1.00	1.55	0.30 ± 0.46	0.00	•
58602	3	10, 10, 20	0.33	0.49	0.13 ± 0.16	0.00	•
59102	2	50, 15	1.00	1.47	0.20 ± 0.28	0.00	•
60002	3	20, 20, 40	0.67	0.85	0.21 ± 0.20	0.00	•
64702	2	40, 25	0.50	0.66	0.27 ± 0.28	0.00	•
75202	4	30, 10, 50, 50	0.50	0.40	0.34 ± 0.14	0.00	•
80702	3	35, 50, 15	0.67	1.08	0.27 ± 0.22	0.00	•
85202	2	10, 15	1.00	0.88	0.20 ± 0.24	0.00	•
93402	2	20, 15	0.50	1.55	0.37 ± 0.31	0.01	•
93902	3	50, 50, 15	0.67	1.85	0.26 ± 0.27	0.00	•
94402	4	10, 35, 30, 10	0.25	0.83	0.21 ± 0.17	0.10	
95202	4	10, 40, 45, 25	0.75	0.42	0.12 ± 0.12	0.00	•
96002	4	25, 40, 35, 45	0.50	0.26	0.13 ± 0.14	0.00	•
98102	2	25, 35	0.50	0.50	0.12 ± 0.25	0.00	•
101702	2	35, 50	1.00	0.34	0.32 ± 0.27	0.00	•
102202	4	50, 45, 50, 50	0.75	0.22	0.07 ± 0.14	0.00	•
104602	2	25, 45	1.00	0.38	0.23 ± 0.31	0.00	•
109502	1	20	0.00	0.00	0.00 ± 0.00	-	
112802	3	10, 15, 15	0.67	1.15	0.18 ± 0.22	0.00	•
113902	3	50, 10, 40	0.33	0.55	0.21 ± 0.22	0.00	•
114702	5	40, 30, 15, 45, 50	0.60	0.62	0.33 ± 0.17	0.00	•
114902	4	25, 50, 50, 20	0.50	0.37	0.16 ± 0.18	0.00	•
123902	2	50, 35	0.50	0.18	0.08 ± 0.19	0.00	•
Overall	-	-	0.64 ± 0.31	3.73 ± 15.82	0.23 ± 0.48	-	32

Even though the sensitivity was above the one from the Surrogate, it was not considered as statistically validated as the FPR/h is extremely large (•).

Table 13. Testing parameters and performance obtained for each patient with the Dynamic Weighted Ensemble and the Add-One-Forget-One data partitioning and iterative retraining method.

Add-One-Forget-One - Dynamic Weighted Ensemble							
**Patient**	**Evaluated seizures**	**SOP**	**SS**	**FPR/h**	**SS Surrogate**	**p-value**	**Above chance**
402	2	10, 15	1.00	2.77	0.25 ± 0.28	0.00	•
8902	2	15, 20	1.00	0.55	0.17 ± 0.24	0.00	•
11002	1	10	0.00	14.37	0.43 ± 0.50	1.00	
16202	4	45, 15, 10, 30	0.75	0.52	0.15 ± 0.15	0.00	•
21902	1	40	0.00	1.50	0.37 ± 0.48	1.00	
23902	2	50, 10	1.00	1.39	0.28 ± 0.28	0.00	•
26102	1	50	1.00	0.88	0.33 ± 0.47	0.00	•
30802	5	35, 15, 20, 25, 50	0.80	0.52	0.19 ± 0.21	0.00	•
32702	2	15, 20	1.00	1.80	0.20 ± 0.28	0.00	•
45402	1	50	1.00	0.00	0.00 ± 0.00	0.00	•
46702	2	30, 10	0.00	0.88	0.13 ± 0.26	1.00	
50802	2	15, 20	1.00	1.27	0.12 ± 0.21	0.00	•
53402	1	15	1.00	2.03	0.20 ± 0.40	0.00	•
55202	5	10, 10, 10, 10, 50	1.00	1.67	0.32 ± 0.15	0.00	•
56402	1	25	1.00	3.30	0.50 ± 0.50	0.00	•
58602	3	10, 10, 20	0.67	0.63	0.16 ± 0.19	0.00	•
59102	2	50, 50	1.00	1.21	0.43 ± 0.33	0.00	•
60002	3	25, 25, 50	1.00	1.06	0.27 ± 0.18	0.00	•
64702	2	50, 25	1.00	2.00	0.53 ± 0.36	0.00	•
75202	4	30, 10, 10, 10	0.50	0.98	0.23 ± 0.21	0.00	•
80702	3	30, 30, 45	1.00	1.19	0.52 ± 0.24	0.00	•
85202	2	10, 15	0.50	1.16	0.17 ± 0.27	0.00	•
93402	2	20, 15	0.50	1.16	0.35 ± 0.29	0.00	•
93902	3	50, 50, 40	0.67	2.54	0.53 ± 0.22	0.00	•
94402	4	10, 50, 25, 10	0.75	0.86	0.23 ± 0.13	0.00	•
95202	4	10, 40, 35, 25	1.00	1.21	0.36 ± 0.26	0.00	•
96002	4	10, 40, 50, 45	0.75	0.58	0.30 ± 0.18	0.00	•
98102	2	25, 50	1.00	0.96	0.25 ± 0.31	0.00	•
101702	2	10, 50	0.50	1.84	0.33 ± 0.3	0.00	•
102202	4	45, 25, 50, 50	0.50	0.18	0.08 ± 0.11	0.00	•
104602	2	20, 30	0.00	1.74	0.37 ± 0.41	1.00	
109502	1	30	0.00	0.00	0.00 ± 0.00	-	
112802	3	10, 10, 30	0.33	2.89	0.28 ± 0.23	0.10	
113902	3	15, 15, 10	0.67	1.31	0.22 ± 0.22	0.00	•
114702	5	50, 30, 10, 45, 50	0.60	0.52	0.26 ± 0.13	0.00	•
114902	4	25, 45, 35, 35	1.00	0.59	0.31 ± 0.19	0.00	•
123902	2	10, 20	0.00	0.98	0.05 ± 0.15	0.96	•
Overall	-	-	0.69 ± 0.36	1.60 ± 2.26	0.25 ± 0.50	-	31

Table 14. Training parameters and performance obtained for each patient with the Backwards-Landmark Window and the Chronological Accumulation data partitioning and iterative retraining method.

Chronological Accumulation - Backwards-Landmark Window

Patient	SOP	k	C	SS_{sample}	SP_{sample}
402	40, 20	10, 30	$2^{-10},2^{0}$	0.53, 0.42	0.62, 0.65
8902	20, 30	10, 10	$2^{-10},2^{0}$	0.82, 0.97	0.78, 0.78
11002	10	20	2^{-10}	0.42	0.71
16202	15, 10, 50, 20	30, 30, 10, 30	$2^{0},2^{0},2^{-10},2^{0}$	0.63, 0.42, 0.52, 0.24	0.82, 0.73, 0.79, 0.71
21902	40	10	2^{6}	0.71	0.73
23902	50, 10	40, 40	$2^{-10},2^{-10}$	0.37, 0.55	0.62, 0.67
26102	50	30	2^{0}	0.31	0.59
30802	10, 10, 50, 25, 50	40, 10, 40, 20, 40	$2^{-10},2^{-10},2^{2},2^{-10},2^{-10}$	0.59, 0.26, 0.45, 0.59, 0.65	0.84, 0.86, 0.53, 0.70, 0.63
32702	15, 25	10, 20	$2^{-10},2^{-10}$	0.57, 0.61	0.74, 0.71
45402	15	30	2^{-10}	0.72	0.53
46702	30, 10	20, 10	$2^{-8},2^{-10}$	0.37, 0.51	0.76, 0.85
50802	15, 25	30, 20	$2^{-8},2^{-10}$	0.70, 0.39	0.86, 0.90
53402	40	10	2^{-10}	0.47	0.65
55202	35, 10, 25, 10, 10	10, 20, 30, 10, 30	$2^{-6},2^{-10},2^{2},2^{-10},2^{-6}$	0.62, 0.51, 0.60, 0.94, 0.53	0.53, 0.72, 0.56, 0.67, 0.69
56402	25	10	2^{-8}	0.41	0.74
58602	10, 10, 10	10, 30, 30	$2^{0},2^{0},2^{0}$	0.24, 0.33, 0.53	0.71, 0.79, 0.75
59102	20, 15	20, 40	$2^{-10},2^{-4}$	0.48, 0.48	0.59, 0.37
60002	20, 20, 40	10, 10, 40	$2^{-8},2^{-8},2^{-10}$	0.44, 0.26, 0.44	0.77, 0.77, 0.83
64702	30, 20	30, 10	$2^{-10},2^{-10}$	0.42, 0.80	0.68, 0.67
75202	30, 10, 45, 35	10, 10, 10, 30	$2^{-4},2^{-10},2^{-10},2^{-4}$	0.68, 0.53, 0.31, 0.56	0.85, 0.83, 0.85, 0.49
80702	35, 50, 15	40, 40, 30	$2^{-10},2^{-8},2^{-10}$	0.3, 0.63, 0.53	0.79, 0.55, 0.51
85202	15, 40	30, 10	$2^{0},2^{0}$	0.45, 0.63	0.66, 0.67
93402	15, 20	20, 40	$2^{4},2^{-6}$	0.45, 0.67	0.71, 0.71
93902	50, 50, 15	10, 20, 20	$2^{-10},2^{-10},2^{-8}$	0.56, 0.45, 0.43	0.62, 0.84, 0.75
94402	10, 40, 15, 15	30, 30, 30, 10	$2^{0},2^{0},2^{0},2^{-10}$	0.41, 0.23, 0.13, 0.54	0.63, 0.64, 0.77, 0.84
95202	10, 35, 40, 10	10, 30, 40, 10	$2^{-10},2^{4},2^{0},2^{-10}$	0.82, 0.35, 0.39, 0.76	0.57, 0.66, 0.68, 0.9
96002	25, 40, 35, 45	20, 30, 40, 20	$2^{-2},2^{-6},2^{-6},2^{8}$	0.52, 0.29, 0.61, 0.39	0.70, 0.63, 0.73, 0.86
98102	35, 45	10, 10	$2^{0},2^{-10}$	0.51, 0.60	0.52, 0.68
101702	30, 50	30, 40	$2^{-4},2^{-2}$	0.63, 0.70	0.51, 0.58
102202	50, 45, 50, 50	30, 40, 30, 10	$2^{-6},2^{-4},2^{-2},2^{-6}$	0.06, 0.32, 0.27, 0.09	0.97, 0.48, 0.48, 0.75
104602	25, 50	10, 30	$2^{-10},2^{0}$	0.43, 0.60	0.62, 0.67
109502	10	10	2^{-10}	0.37	0.79
112802	10, 15, 15	10, 10, 40	$2^{-10},2^{-6},2^{-10}$	0.65, 0.38, 0.19	0.49, 0.58, 0.79
113902	45, 15, 45	30, 30, 10	$2^{-10},2^{-10},2^{-10}$	0.42, 0.64, 0.46	0.56, 0.73, 0.61
114702	35, 15, 15, 50, 15	30, 40, 40, 10, 30	$2^{-10},2^{-4},2^{-10},2^{-10},2^{-10}$	0.22, 0.17, 0.67, 0.41, 0.56	0.76, 0.93, 0.70, 0.53, 0.74
114902	25, 50, 50, 20	20, 30, 10, 10	$2^{0},2^{8},2^{-10},2^{-10}$	0.53, 0.04, 0.15, 0.67	0.44, 0.77, 0.91, 0.84
123902	10, 10	30, 30	$2^{-10},2^{-10}$	0.79, 0.65	0.84, 0.83
Overall	-	-	-	0.49 ± 0.19	0.70 ± 0.12

Table 15. Training parameters and performance obtained for each patient with the Seizure-batch Regression and the Chronological Accumulation data partitioning and iterative retraining method.

Chronological Accumulation - Seizure-batch Regression

Patient	SOP	k	C	SS_{sample}	SP_{sample}
402	10, 15	10, 10	$2^{-10}, 2^{8}$	0.69, 0.19	0.65, 0.59
8902	20, 20	30, 30	$2^{-10}, 2^{-10}$	0.87, 0.91	0.84, 0.85
11002	10	20	2^{-10}	0.53	0.61
16202	45, 15, 10, 30	10, 40, 20, 40	$2^{-10}, 2^{8}, 2^{8}, 2^{6}$	0.83, 0.55, 0.46, 0.21	0.61, 0.59, 0.69, 0.71
21902	10	10	2^{-10}	0.67	0.61
23902	50, 45	40, 10	$2^{-8}, 2^{-10}$	0.50, 0.66	0.53, 0.44
26102	50	40	2^{6}	0.66	0.35
30802	50, 50, 50, 35, 35	30, 10, 10, 10, 10	$2^{-10}, 2^{0}, 2^{-10}, 2^{0}, 2^{-10}$	0.90, 0.72, 0.76, 0.79, 0.79	0.79, 0.78, 0.65, 0.66, 0.59
32702	15, 15	10, 10	$2^{-2}, 2^{-10}$	0.67, 0.81	0.70, 0.63
45402	50	40	2^{-10}	0.67	0.55
46702	35, 25	30, 30	$2^{0}, 2^{0}$	0.20, 0.49	0.68, 0.63
50802	15, 20	30, 30	$2^{-10}, 2^{-10}$	0.82, 0.73	0.70, 0.8
53402	15	20	2^{-4}	0.22	0.72
55202	10, 10, 10, 10, 10	30, 10, 30, 30, 30	$2^{0}, 2^{-10}, 2^{-10}, 2^{-10}, 2^{-10}$	0.50, 0.74, 0.69, 0.67, 0.71	0.73, 0.67, 0.55, 0.64, 0.61
56402	25	20	2^{-10}	0.46	0.72
58602	10, 10, 20	40, 40, 40	$2^{-10}, 2^{-10}, 2^{-8}$	0.15, 0.17, 0.65	0.78, 0.87, 0.73
59102	15, 50	10, 30	$2^{-10}, 2^{0}$	0.66, 0.52	0.44, 0.43
60002	25, 15, 15	30, 20, 20	$2^{-10}, 2^{-10}, 2^{-8}$	0.56, 0.44, 0.44	0.65, 0.66, 0.7
64702	50, 25	30, 30	$2^{-6}, 2^{-6}$	0.64, 0.71	0.46, 0.55
75202	30, 10, 30, 40	30, 10, 30, 10	$2^{-10}, 2^{-10}, 2^{0}, 2^{-10}$	0.71, 0.73, 0.69, 0.68	0.83, 0.82, 0.62, 0.61
80702	30, 30, 50	20, 10, 30	$2^{-10}, 2^{-10}, 2^{-6}$	0.51, 0.63, 0.61	0.63, 0.45, 0.43
85202	10, 15	30, 20	$2^{6}, 2^{8}$	0.25, 0.65	0.79, 0.53
93402	50, 20	10, 30	$2^{-10}, 2^{0}$	0.57, 0.44	0.52, 0.75
93902	50, 50, 40	30, 30, 10	$2^{-10}, 2^{-10}, 2^{-10}$	0.71, 0.75, 0.46	0.51, 0.61, 0.68
94402	10, 50, 25, 10	10, 10, 30, 40	$2^{4}, 2^{-10}, 2^{-6}, 2^{-10}$	0.48, 0.82, 0.29, 0.49	0.62, 0.36, 0.71, 0.86
95202	10, 30, 10, 40	10, 30, 10, 30	$2^{-10}, 2^{0}, 2^{-10}, 2^{0}$	0.74, 0.28, 0.68, 0.55	0.66, 0.62, 0.66, 0.55
96002	10, 20, 30, 30	40, 40, 30, 30	$2^{-10}, 2^{-10}, 2^{-10}, 2^{-10}$	0.84, 0.46, 0.58, 0.56	0.56, 0.67, 0.57, 0.60
98102	25, 50	40, 20	$2^{8}, 2^{-2}$	0.58, 0.63	0.32, 0.58
101702	10, 40	10, 10	$2^{0}, 2^{0}$	0.49, 0.60	0.60, 0.51
102202	45, 25, 50, 50	30, 40, 30, 10	$2^{0}, 2^{6}, 2^{4}, 2^{4}$	0.12, 0.25, 0.35, 0.24	0.88, 0.78, 0.59, 0.72
104602	20, 30	10, 10	$2^{-10}, 2^{-10}$	0.51, 0.8	0.58, 0.60
109502	10	10	2^{-10}	0.56	0.54
112802	10, 15, 15	10, 10, 10	$2^{-10}, 2^{8}, 2^{8}$	0.77, 0.49, 0.42	0.37, 0.42, 0.5
113902	15, 15, 10	20, 30, 40	$2^{-10}, 2^{-10}, 2^{-10}$	0.35, 0.60, 0.59	0.65, 0.76, 0.64
114702	35, 45, 10, 15, 15	10, 10, 30, 30, 10	$2^{0}, 2^{0}, 2^{0}, 2^{0}, 2^{0}$	0.25, 0.36, 0.44, 0.4, 0.57	0.73, 0.55, 0.63, 0.63, 0.53
114902	25, 35, 35, 35	10, 10, 10, 40	$2^{-10}, 2^{-10}, 2^{-10}, 2^{-10}$	0.70, 0.31, 0.31, 0.57	0.23, 0.60, 0.71, 0.66
123902	10, 20	40, 10	$2^{-6}, 2^{-4}$	0.92, 0.31	0.80, 0.88
Overall	-	-	-	0.56 ± 0.19	0.63 ± 0.13

Table 16. Training parameters and performance obtained for each patient with the Dynamic Weighted Ensemble and the Chronological Accumulation data partitioning and iterative retraining method.

Chronological Accumulation - Dynamic Weighted Ensemble

Patient	SOP	k	C	SS_{sample}	SP_{sample}
402	10, 50	10, 40	$2^{-10}, 2^{6}$	0.69, 0.32	0.65, 0.68
8902	15, 20	10, 10	$2^{0}, 2^{4}$	0.90, 0.95	0.68, 0.80
11002	10	20	2^{-10}	0.53	0.61
16202	45, 15, 15, 50	10, 30, 30, 40	$2^{-10}, 2^{2}, 2^{2}, 2^{6}$	0.83, 0.47, 0.43, 0.45	0.61, 0.70, 0.70, 0.69
21902	40	40	2^{-10}	0.84	0.58
23902	50, 45	40, 10	$2^{-8}, 2^{-10}$	0.50, 0.66	0.53, 0.44
26102	50	40	2^{6}	0.66	0.35
30802	35, 10, 10, 25, 50	40, 20, 20, 40, 10	$2^{-8}, 2^{-10}, 2^{-10}, 2^{-10}, 2^{-10}$	0.92, 0.58, 0.75, 0.65, 0.76	0.78, 0.83, 0.53, 0.68, 0.52
32702	15, 15	10, 10	$2^{-2}, 2^{-10}$	0.67, 0.81	0.70, 0.63
45402	50	40	2^{-10}	0.67	0.55
46702	30, 10	40, 40	$2^{-2}, 2^{8}$	0.37, 0.59	0.69, 0.69
50802	15, 20	30, 30	$2^{-10}, 2^{-10}$	0.82, 0.73	0.70, 0.8
53402	15	20	2^{-4}	0.22	0.72
55202	10, 10, 10, 10, 10	10, 10, 10, 10, 10	$2^{-10}, 2^{-10}, 2^{-10}, 2^{-10}, 2^{-8}$	0.84, 0.73, 0.70, 0.76, 0.75	0.52, 0.74, 0.56, 0.62, 0.57
56402	25	20	2^{-10}	0.46	0.72
58602	10, 10, 20	40, 40, 30	$2^{-10}, 2^{-10}, 2^{-10}$	0.15, 0.26, 0.33	0.78, 0.83, 0.76
59102	50, 45	10, 10	$2^{-10}, 2^{-10}$	0.41, 0.70	0.42, 0.26
60002	25, 15, 15	30, 20, 20	$2^{-10}, 2^{-10}, 2^{-8}$	0.56, 0.44, 0.44	0.65, 0.66, 0.70
64702	50, 25	30, 40	$2^{-6}, 2^{-4}$	0.64, 0.64	0.46, 0.55
75202	30, 10, 50, 10	30, 20, 40, 40	$2^{-10}, 2^{-10}, 2^{-8}, 2^{-6}$	0.82, 0.64, 0.68, 0.54	0.75, 0.82, 0.74, 0.69
80702	30, 30, 50	20, 10, 30	$2^{-10}, 2^{-10}, 2^{-6}$	0.51, 0.63, 0.61	0.63, 0.45, 0.43
85202	10, 15	30, 40	$2^{6}, 2^{2}$	0.25, 0.28	0.79, 0.76
93402	20, 15	10, 10	$2^{-10}, 2^{8}$	0.64, 0.63	0.43, 0.48
93902	50, 50, 40	30, 30, 10	$2^{-10}, 2^{-10}, 2^{-10}$	0.71, 0.75, 0.46	0.51, 0.61, 0.68
94402	10, 10, 10, 10	10, 40, 40, 40	$2^{4}, 2^{-10}, 2^{-10}, 2^{-8}$	0.48, 0.71, 0.53, 0.57	0.62, 0.42, 0.51, 0.56
95202	10, 40, 40, 40	10, 40, 40, 40	$2^{-10}, 2^{6}, 2^{2}, 2^{2}$	0.77, 0.56, 0.59, 0.68	0.43, 0.43, 0.36, 0.42
96002	10, 20, 30, 30	40, 40, 30, 30	$2^{-10}, 2^{-10}, 2^{-10}, 2^{-10}$	0.84, 0.46, 0.58, 0.56	0.56, 0.67, 0.57, 0.6
98102	25, 50	40, 40	$2^{8}, 2^{8}$	0.58, 0.69	0.32, 0.54
101702	10, 50	20, 30	$2^{-6}, 2^{-6}$	0.50, 0.46	0.57, 0.55
102202	45, 25, 50, 50	30, 40, 30, 10	$2^{0}, 2^{6}, 2^{4}, 2^{4}$	0.12, 0.25, 0.35, 0.24	0.88, 0.78, 0.59, 0.72
104602	20, 25	10, 10	$2^{-10}, 2^{-10}$	0.51, 0.54	0.58, 0.61
109502	30	10	2^{-6}	0.34	0.71
112802	10, 15, 15	10, 10, 10	$2^{-10}, 2^{8}, 2^{8}$	0.77, 0.49, 0.42	0.37, 0.42, 0.5
113902	15, 15, 15	20, 20, 20	$2^{-10}, 2^{-10}, 2^{-10}$	0.35, 0.44, 0.41	0.65, 0.75, 0.68
114702	50, 30, 30, 45, 50	30, 40, 40, 40, 40	$2^{-8}, 2^{4}, 2^{-10}, 2^{4}, 2^{4}$	0.46, 0.24, 0.28, 0.36, 0.44	0.41, 0.61, 0.64, 0.61, 0.57
114902	25, 35, 35, 35	10, 10, 10, 40	$2^{-10}, 2^{-10}, 2^{-10}, 2^{-10}$	0.7, 0.31, 0.31, 0.57	0.23, 0.6, 0.71, 0.66
123902	10, 10	40, 40	$2^{-6}, 2^{-10}$	0.92, 0.46	0.80, 0.88
Overall	-	-	-	0.56 ± 0.19	0.61 ± 0.14

Table 17. Testing parameters and performance obtained for each patient with the Backwards-Landmark Window and the Chronological Accumulation data partitioning and iterative retraining method.

Chronological Accumulation - Backwards-Landmark Window

Patient	Evaluated seizures	SOP	SS	FPR/h	SS Surrogate	p-value	Above chance
402	2	40, 20	0.50	1.85	0.32 ± 0.24	0.00	•
8902	2	20, 30	0.50	0.19	0.12 ± 0.21	0.00	•
11002	1	10	1.00	0.00	0.00 ± 0.00	0.00	•
16202	4	15, 10, 50, 20	0.75	6.98	0.51 ± 0.21	0.00	•
21902	1	40	1.00	1.23	0.47 ± 0.50	0.00	•
23902	2	50, 10	1.00	2.74	0.58 ± 0.34	0.00	•
26102	1	50	1.00	2.88	0.67 ± 0.47	0.00	•
30802	5	10, 10, 50, 25, 50	0.20	1.51	0.27 ± 0.21	0.97	
32702	2	15, 25	0.50	1.67	0.17 ± 0.24	0.00	•
45402	1	15	1.00	0.23	0.13 ± 0.34	0.00	•
46702	2	30, 10	0.50	3.54	0.48 ± 0.38	0.41	
50802	2	15, 25	0.50	0.90	0.10 ± 0.24	0.00	•
53402	1	40	1.00	0.03	0.07 ± 0.25	0.00	•
55202	5	35, 10, 25, 10, 10	0.20	0.29	0.15 ± 0.14	0.02	•
56402	1	25	0.00	4.77	0.50 ± 0.50	1.00	
58602	3	10, 10, 10	0.33	0.71	0.06 ± 0.12	0.00	•
59102	2	20, 15	1.00	1.00	0.38 ± 0.21	0.00	•
60002	3	20, 20, 40	0.33	0.28	0.14 ± 0.21	0.00	•
64702	2	30, 20	0.50	0.52	0.35 ± 0.39	0.02	•
75202	4	30, 10, 45, 35	0.25	0.02	0.01 ± 0.04	0.00	•
80702	3	35, 50, 15	0.67	2.17	0.16 ± 0.17	0.00	•
85202	2	15, 40	0.50	9.62	0.37 ± 0.29	0.01	•
93402	2	15, 20	0.50	0.31	0.03 ± 0.12	0.00	•
93902	3	50, 50, 15	0.33	1.91	0.54 ± 0.25	1.00	
94402	4	10, 40, 15, 15	0.50	2.50	0.20 ± 0.22	0.00	•
95202	4	10, 35, 40, 10	0.25	1.10	0.18 ± 0.2	0.04	•
96002	4	25, 40, 35, 45	0.25	1.50	0.51 ± 0.19	1.00	
98102	2	35, 45	1.00	12.98	0.65 ± 0.37	0.00	•
101702	2	30, 50	0.50	0.07	0.05 ± 0.15	0.00	•
102202	4	50, 45, 50, 50	0.00	0.32	0.08 ± 0.11	1.00	
104602	2	25, 50	1.00	0.74	0.23 ± 0.25	0.00	•
109502	1	10	0.00	0.47	0.03 ± 0.18	0.84	
112802	3	10, 15, 15	0.33	1.09	0.28 ± 0.24	0.12	
113902	3	45, 15, 45	1.00	1.36	0.42 ± 0.21	0.00	•
114702	5	35, 15, 15, 50, 15	0.00	0.16	0.05 ± 0.09	1.00	
114902	4	25, 50, 50, 20	0.25	0.03	0.03 ± 0.08	0.00	•
123902	2	10, 10	0.50	0.14	0.03 ± 0.12	0.00	•
Overall	-	-	0.53 ± 0.33	1.83 ± 2.71	0.23 ± 0.48	-	28

Table 18. Testing parameters and performance obtained for each patient with the Seizure-batch Regression and the Chronological Accumulation data partitioning and iterative retraining method.

Chronological Accumulation - Seizure-batch Regression

Patient	Evaluated seizures	SOP	SS	FPR/h	SS Surrogate	p-value	Above chance
402	2	10, 15	0.00	9.38	0.25 ± 0.34	1.00	
8902	2	20, 20	0.50	0.31	0.22 ± 0.25	0.00	•
11002	1	10	0.00	11.96	0.37 ± 0.48	1.00	
16202	4	45, 15, 10, 30	0.00	0.40	0.13 ± 0.15	1.00	
21902	1	10	1.00	0.19	0.30 ± 0.46	0.00	•
23902	2	50, 45	1.00	1.17	0.33 ± 0.32	0.00	•
26102	1	50	1.00	0.05	0.10 ± 0.30	0.00	•
30802	5	50, 50, 50, 35, 35	0.80	0.61	0.39 ± 0.17	0.00	•
32702	2	15, 15	0.50	1.63	0.15 ± 0.23	0.00	•
45402	1	50	1.00	97.86	0.63 ± 0.48	0.00	•
46702	2	35, 25	0.50	0.52	0.22 ± 0.31	0.00	•
50802	2	15, 20	0.50	0.39	0.07 ± 0.17	0.00	•
53402	1	15	1.00	0.03	0.03 ± 0.18	0.00	•
55202	5	10, 10, 10, 10, 10	0.20	1.18	0.19 ± 0.18	0.35	
56402	1	25	1.00	1.55	0.27 ± 0.44	0.00	•
58602	3	10, 10, 20	0.67	0.22	0.03 ± 0.10	0.00	•
59102	2	15, 50	0.50	1.20	0.57 ± 0.33	0.85	
60002	3	25, 15, 15	0.33	0.64	0.19 ± 0.25	0.00	•
64702	2	50, 25	0.50	0.43	0.20 ± 0.24	0.00	•
75202	4	30, 10, 30, 40	0.00	0.10	0.05 ± 0.1	0.99	
80702	3	30, 30, 50	0.67	0.60	0.23 ± 0.20	0.00	•
85202	2	10, 15	0.50	0.50	0.08 ± 0.19	0.00	•
93402	2	50, 20	1.00	1.58	0.48 ± 0.33	0.00	•
93902	3	50, 50, 40	0.33	1.51	0.24 ± 0.23	0.02	•
94402	4	10, 50, 25, 10	0.25	5.11	0.35 ± 0.25	0.98	
95202	4	10, 30, 10, 40	0.25	0.41	0.06 ± 0.12	0.00	•
96002	4	10, 20, 30, 30	0.00	2.68	0.43 ± 0.23	1.00	
98102	2	25, 50	0.50	0.55	0.15 ± 0.29	0.00	•
101702	2	10, 40	0.50	0.24	0.20 ± 0.24	0.00	•
102202	4	45, 25, 50, 50	1.00	0.83	0.38 ± 0.19	0.00	•
104602	2	20, 30	0.50	0.82	0.28 ± 0.31	0.00	•
109502	1	10	0.00	0.00	0.00 ± 0.00	-	
112802	3	10, 15, 15	0.33	3.01	0.29 ± 0.21	0.13	
113902	3	15, 15, 10	1.00	2.39	0.72 ± 0.21	0.00	•
114702	5	35, 45, 10, 15, 15	0.00	0.17	0.07 ± 0.09	1.00	
114902	4	25, 35, 35, 35	0.00	0.21	0.13 ± 0.14	1.00	
123902	2	10, 20	0.50	0.18	0.05 ± 0.15	0.00	•
Overall	-	-	0.50 ± 0.36	4.07 ± 15.82	0.23 ± 0.49	-	24

Even though the sensitivity was above the one from the Surrogate, it was not considered as statistically validated as the FPR/h is extremely large (•).

Table 19. Testing parameters and performance obtained for each patient with the Dynamic Weighted Ensemble and the Chronological Accumulation data partitioning and iterative retraining method.

Chronological Accumulation - Dynamic Weighted Ensemble

Patient	Evaluated seizures	SOP	SS	FPR/h	SS Surrogate	p-value	Above chance
402	2	10, 50	1.00	2.23	0.20 ± 0.24	0.00	•
8902	2	15, 20	0.50	0.54	0.18 ± 0.27	0.00	•
11002	1	10	0.00	14.37	0.33 ± 0.47	1.00	
16202	4	45, 15, 15, 50	0.50	0.77	0.21 ± 0.18	0.00	•
21902	1	40	0.00	1.50	0.40 ± 0.49	1.00	
23902	2	50, 45	1.00	2.59	0.50 ± 0.34	0.00	•
26102	1	50	1.00	0.88	0.47 ± 0.50	0.00	•
30802	5	35, 10, 10, 25, 50	0.80	1.45	0.32 ± 0.14	0.00	
32702	2	15, 15	0.50	1.86	0.27 ± 0.31	0.00	•
45402	1	50	1.00	0.00	0.00 ± 0.00	0.00	•
46702	2	30, 10	0.00	0.88	0.13 ± 0.22	1.00	
50802	2	15, 20	1.00	1.21	0.15 ± 0.23	0.00	•
53402	1	15	1.00	2.03	0.27 ± 0.44	0.00	•
55202	5	10, 10, 10, 10, 10	0.60	2.21	0.22 ± 0.16	0.00	•
56402	1	25	1.00	3.30	0.43 ± 0.50	0.00	
58602	3	10, 10, 20	0.33	0.48	0.08 ± 0.14	0.00	•
59102	2	50, 45	1.00	1.23	0.47 ± 0.31	0.00	•
60002	3	25, 15, 15	0.67	1.48	0.23 ± 0.21	0.00	•
64702	2	50, 25	1.00	2.14	0.53 ± 0.31	0.00	•
75202	4	30, 10, 50, 10	0.50	0.41	0.10 ± 0.17	0.00	•
80702	3	30, 30, 50	1.00	1.52	0.52 ± 0.19	0.00	•
85202	2	10, 15	0.00	0.64	0.10 ± 0.24	0.98	
93402	2	20, 15	0.50	2.30	0.45 ± 0.35	0.22	
93902	3	50, 50, 40	0.33	2.05	0.39 ± 0.27	0.86	
94402	4	10, 10, 10, 10	0.75	4.16	0.29 ± 0.21	0.00	•
95202	4	10, 40, 40, 40	1.00	1.99	0.49 ± 0.25	0.00	•
96002	4	10, 20, 30, 30	0.75	2.39	0.39 ± 0.21	0.00	•
98102	2	25, 50	1.00	0.90	0.20 ± 0.31	0.00	•
101702	2	10, 50	0.50	4.05	0.40 ± 0.37	0.08	
102202	4	45, 25, 50, 50	0.50	0.03	0.00 ± 0.00	0.00	•
104602	2	20, 25	0.00	0.79	0.10 ± 0.20	0.99	•
109502	1	30	0.00	0.00	0.00 ± 0.00	-	
112802	3	10, 15, 15	0.67	2.03	0.26 ± 0.22	0.00	•
113902	3	15, 15, 15	0.67	1.31	0.23 ± 0.21	0.00	•
114702	5	50, 30, 30, 45, 50	0.80	0.86	0.44 ± 0.2.0	0.00	•
114902	4	25, 35, 35, 35	0.75	0.48	0.26 ± 0.18	0.00	•
123902	2	10, 10	0.00	0.97	0.07 ± 0.17	0.98	•
Overall	-	-	0.61 ± 0.36	1.84 ± 2.31	0.25 ± 0.50	-	28

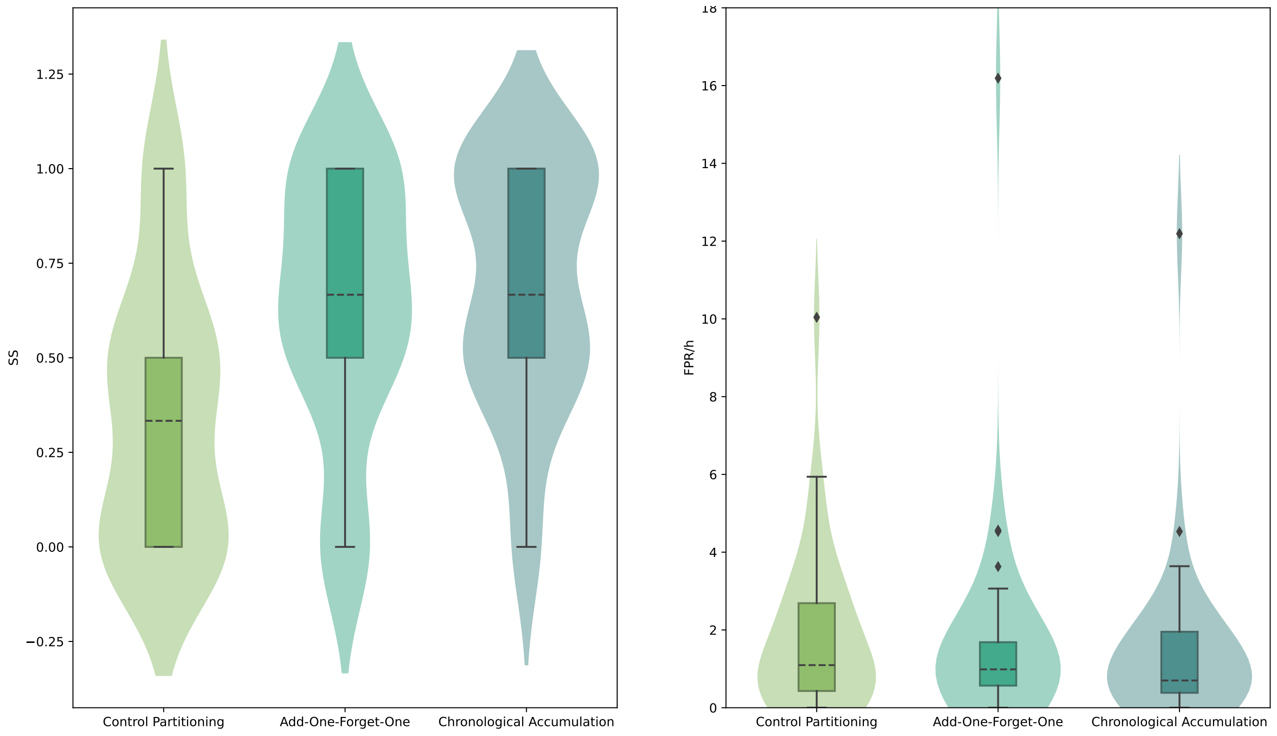

Figure 1. Seizure prediction performance across all patients for each data partitioning and iterative retraining method.

Table 20. Average seizure prediction performance across all patients for each approach, both Add-One-Forget-One and Chronological Accumulation methods.

Approach	Retraining method	All patients			Validated patients		
		**SS**	**FPR/h**	**SS Surrogate**	**%**	**SS**	**FPR/h**
Control	Add-One-Forget-One	0.63 ± 0.34	1.72 ± 2.65	0.26 ± 0.51	83.78	0.70 ± 0.27	1.69 ± 2.75
	Chronological Accumulation	0.68 ± 0.31	1.44 ± 2.06	0.23 ± 0.48	91.89	0.70 ± 0.30	1.32 ± 2.07
Backwards-Landmark Window	Add-One-Forget-One	0.75 ± 0.33	1.03 ± 1.00	0.22 ± 0.47	89.19	0.81 ± 0.25	0.91 ± 0.81
	Chronological Accumulation	0.53 ± 0.33	1.83 ± 2.71	0.23 ± 0.48	75.68	0.66 ± 0.29	1.92 ± 3.05
Seizure-batch Regression	Add-One-Forget-One	0.64 ± 0.31	3.73 ± 15.82	0.23 ± 0.48	86.49	0.68 ± 0.27	0.67 ± 0.51
	Chronological Accumulation	0.50 ± 0.36	4.07 ± 15.82	0.23 ± 0.49	64.86	0.67 ± 0.26	0.72 ± 0.59
Dynamic Weighted Ensemble	Add-One-Forget-One	0.69 ± 0.36	1.6 ± 2.26	0.25 ± 0.50	83.78	0.79 ± 0.28	1.18 ± 0.76
	Chronological Accumulation	0.61 ± 0.36	1.84 ± 2.31	0.25 ± 0.51	75.68	0.64 ± 0.34	1.49 ± 1.05

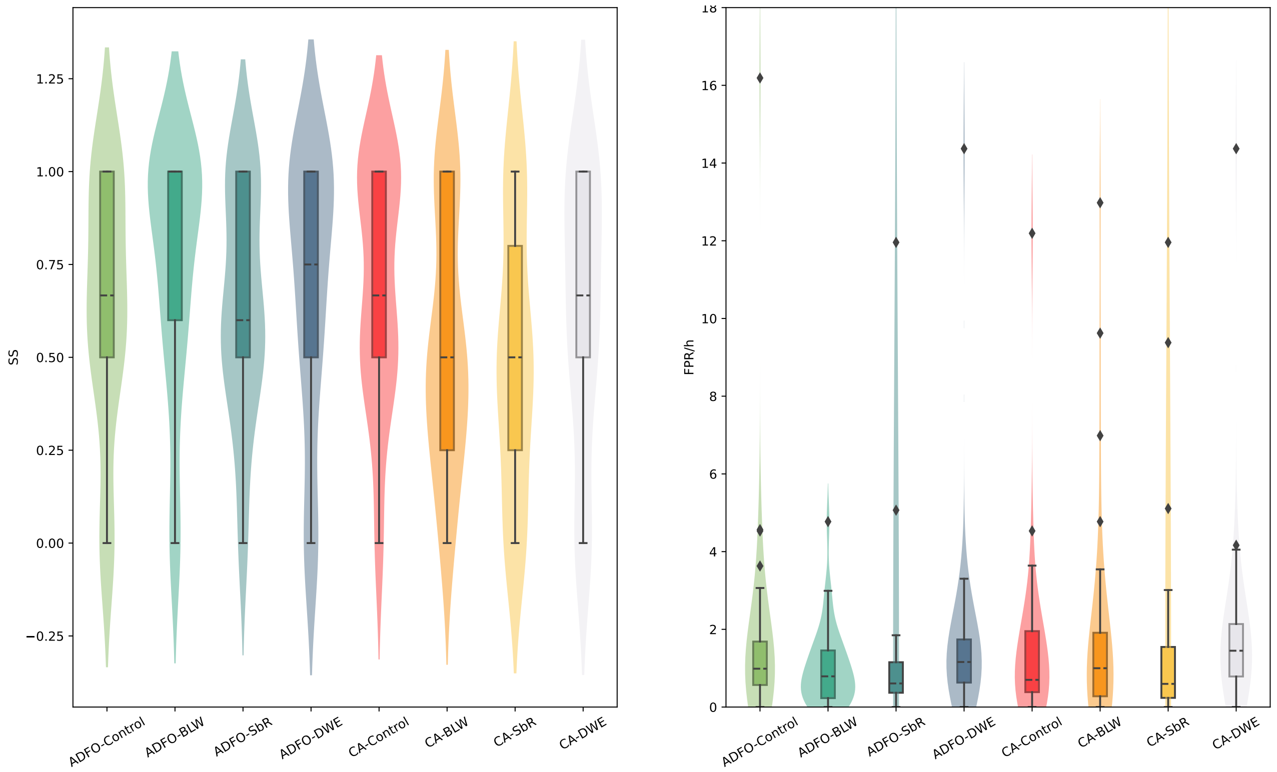

Figure 2. Seizure prediction performance across all patients for each approach, both Add-One-Forget-One and Chronological Accumulation methods. Seizure-batch Regression has an outlier of 97.86 FPR/h for both iterative retraining methods. AOFO stands for Add-One-Forget-One, CA for Chronological Accumulation, BLW for Backwards-Landmark Window, SbR for Seizure-batch Regression, and DWE for Dynamic Weighted Ensemble.

Concept drift adaptation analysis

For patient 55202, as seizures occur, the preictal period tends to a duration of 10 minutes, and the SVM costs are in the majority to the power of -6 and -10. Nonetheless, the algorithm, in addition to never selecting the channels F4 and P8 (see Figure 4), the selected channels overall vary significantly between seizures, we believe this may highlight different seizure generating processes. We noticed for all, except the 6th, evaluated seizures, a predominance of the relative power features of the beta, gamma1, gamma2, and gamma3 bands. Also, we selected some Hjörth parameters. For the 6th seizure in particular, no relative power features were selected, but the ratios between the beta, gamma1, gamma2, and gamma3 bands, were chosen instead. Also, the number of selected features varied between 10 and 30, but there was a preference for 10 and 30.

Finally, for patient 114702, there is also a clear preference for the SVM cost of 2^{-10}; the preictal duration is usually 15 minutes but is once 35 and other time 50 minutes. Regarding the electrode selection, all of them were chosen, ones more frequently than others. In particular, for the 7th seizure, only two electrodes from the temporal lobes were selected (see Figure 5). However, for features other than a predominance for measures related to the theta, beta, gamma1, gamma2, and gamma3 bands, other features were selected, such as Hjörth parameters and wavelets, where five energy levels were kept. Also, the number of features chosen varied between 10 and 40, but there was a preference for 30 and 40.

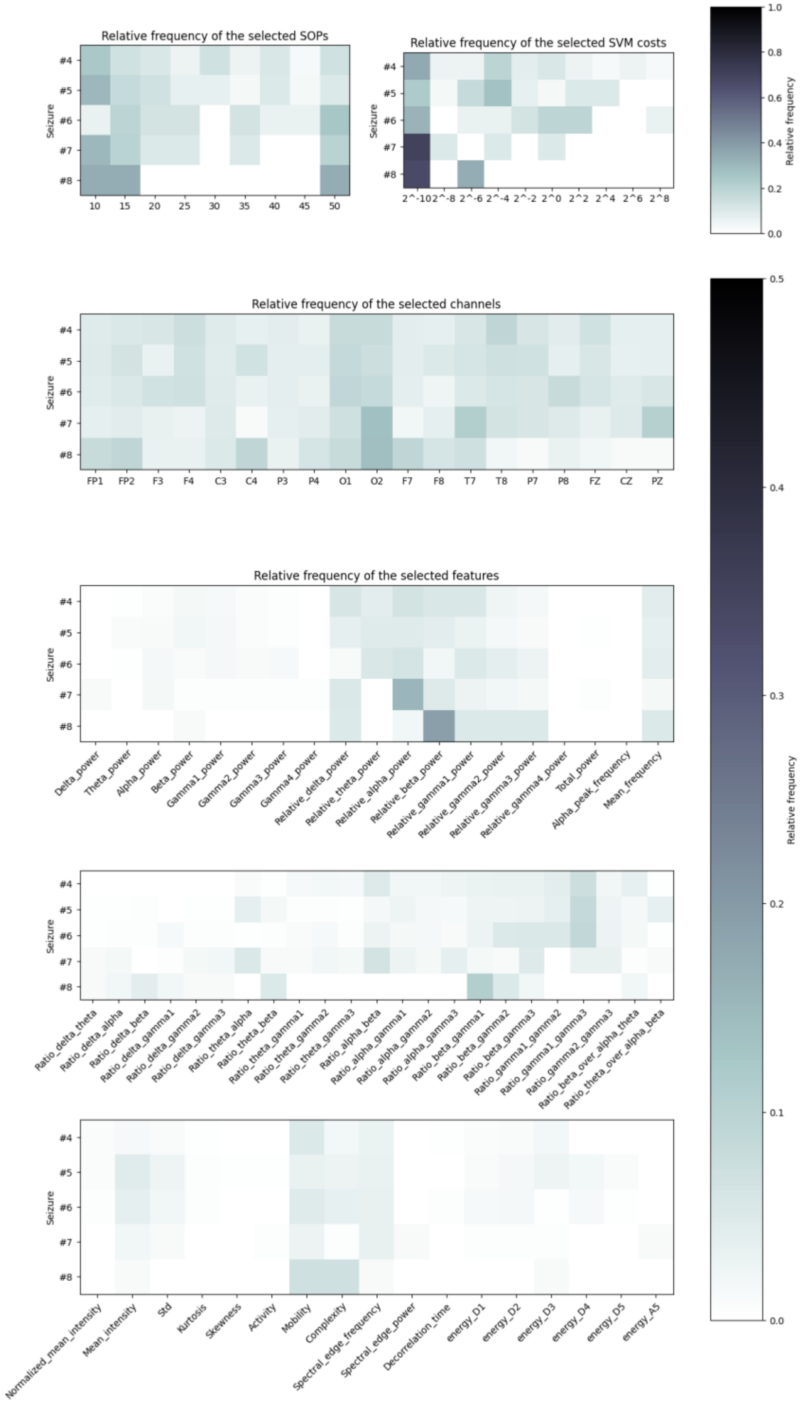

Figure 3. Relative frequency of the selected SOP duration, SVM costs, channels, and features for all 37 patients.

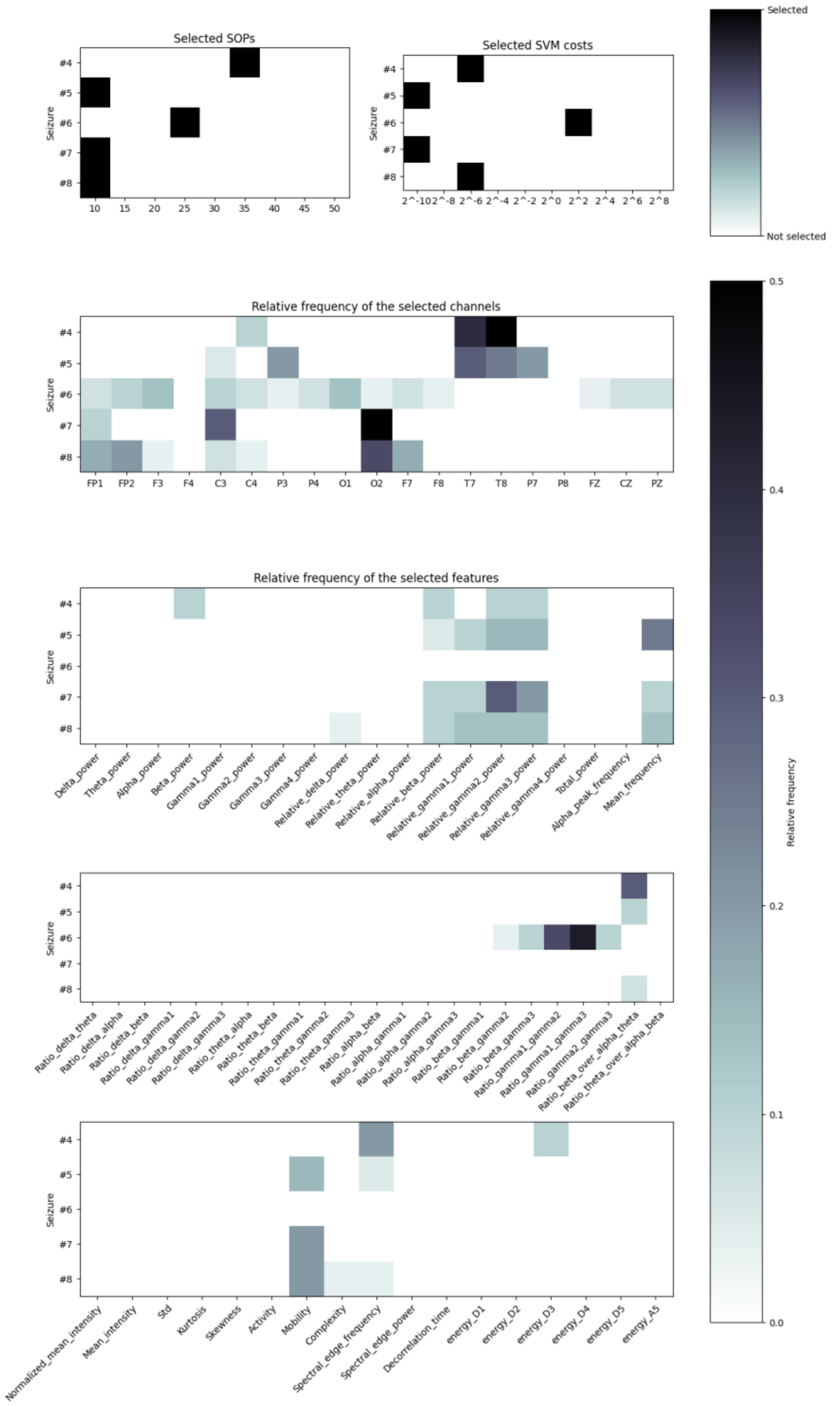

Figure 4. Relative frequency of the selected SOP duration, SVM costs, channels, and features for patient 55202.

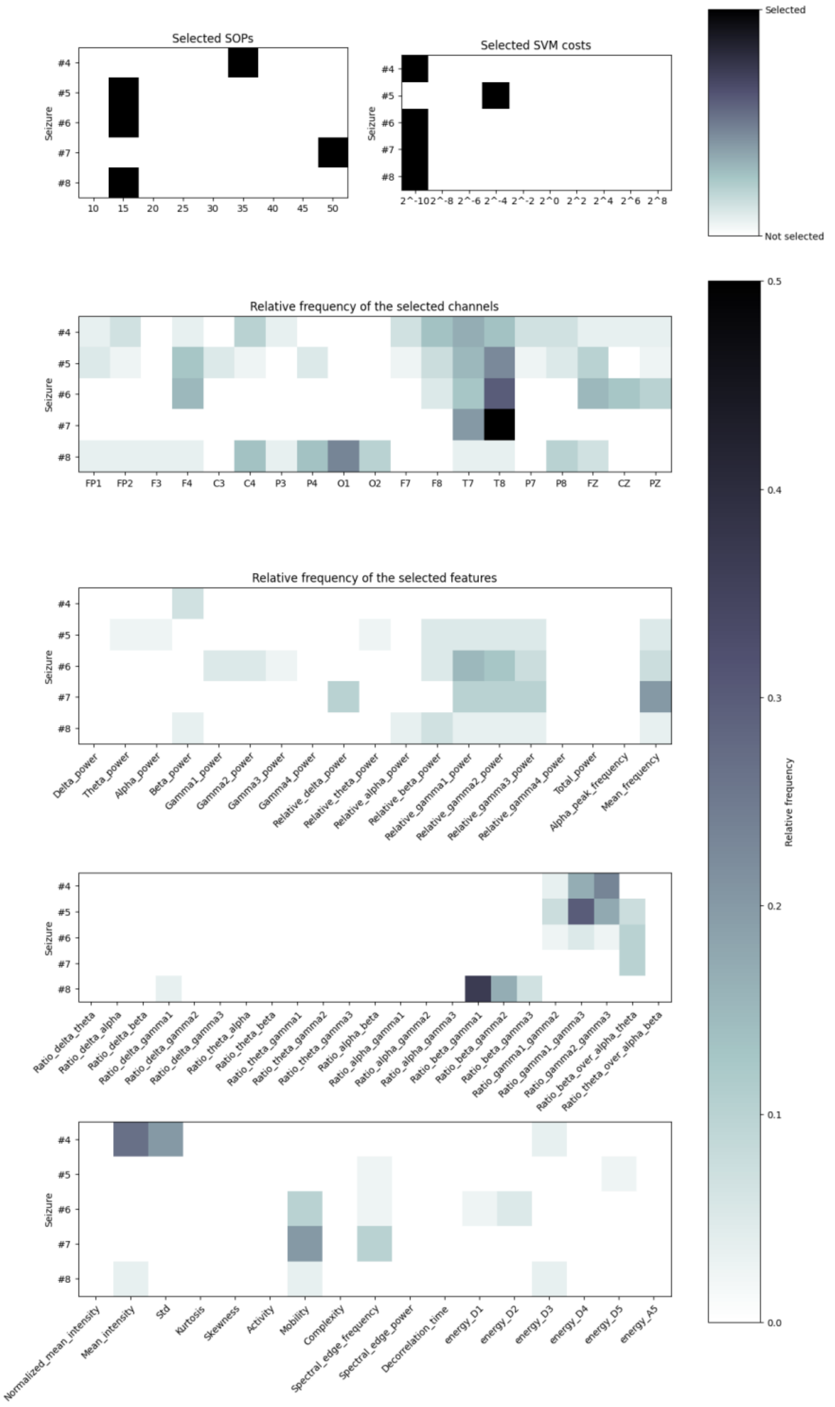

Figure 5. Relative frequency of the selected SOP duration, SVM costs, channels, and features for patient 114702.

References

1. Mormann, F. *et al.* Automated detection of a preseizure state based on a decrease in synchronization in intracranial electroencephalogram recordings from epilepsy patients. *Phys. Rev. E* **67**, 021912 (2003).
2. Kuhlmann, L., Lehnertz, K., Richardson, M. P., Schelter, B. & Zaveri, H. P. Seizure prediction—ready for a new era. *Nat. Rev. Neurol.* **14**, 618–630 (2018).
3. Direito, B., Teixeira, C. A., Sales, F., Castelo-Branco, M. & Dourado, A. A realistic seizure prediction study based on multiclass svm. *Int. journal neural systems* **27**, 1750006 (2017).
4. Rasekhi, J., Mollaei, M. R. K., Bandarabadi, M., Teixeira, C. A. & Dourado, A. Epileptic seizure prediction based on ratio and differential linear univariate features. *J. medical signals sensors* **5**, 1 (2015).
5. Teixeira, C. A. *et al.* Epileptic seizure predictors based on computational intelligence techniques: A comparative study with 278 patients. *Comput. methods programs biomedicine* **114**, 324–336 (2014).
6. Bou Assi, E., Nguyen, D. K., Rihana, S. & Sawan, M. Towards accurate prediction of epileptic seizures: A review. *Biomed. Signal Process. Control.* **34**, 144–157, DOI: https://doi.org/10.1016/j.bspc.2017.02.001 (2017).
7. Mormann, F., Andrzejak, R. G., Elger, C. E. & Lehnertz, K. Seizure prediction: the long and winding road. *Brain* **130**, 314–333 (2007).
8. Rasekhi, J., Mollaei, M. R. K., Bandarabadi, M., Teixeira, C. A. & Dourado, A. Preprocessing effects of 22 linear univariate features on the performance of seizure prediction methods. *J. Neurosci. Methods* **217**, 9–16, DOI: 10.1016/j.jneumeth.2013.03.019 (2013).
9. Moghim, N. & Corne, D. W. Predicting epileptic seizures in advance. *PLoS One* **9**, DOI: 10.1371/journal.pone.0099334 (2014).
10. Mormann, F. *et al.* On the predictability of epileptic seizures. *Clin. neurophysiology* **116**, 569–587 (2005).
11. Assi, E. B., Sawan, M., Nguyen, D. K. & Rihana, S. A hybrid mrmr-genetic based selection method for the prediction of epileptic seizures. In *2015 IEEE Biomedical Circuits and Systems Conference (BioCAS)*, 1–4 (IEEE, 2015).
12. Bandarabadi, M., Teixeira, C. A., Rasekhi, J. & Dourado, A. Epileptic seizure prediction using relative spectral power features. *Clin. Neurophysiol.* **126**, 237–248 (2015).
